# Increased fecal ethanol and enriched ethanol-producing gut bacteria *Limosilactobacillus fermentum*, *Enterocloster bolteae*, *Mediterraneibacter gnavus* and *Streptococcus mutans* in nonalcoholic steatohepatitis

**DOI:** 10.3389/fcimb.2023.1279354

**Published:** 2023-11-16

**Authors:** Babacar Mbaye, Reham Magdy Wasfy, Patrick Borentain, Maryam Tidjani Alou, Giovanna Mottola, Vincent Bossi, Aurelia Caputo, Rene Gerolami, Matthieu Million

**Affiliations:** ^1^ Aix-Marseille Université, IRD, APHM, MEPHI, IHU Méditerranée Infection, Marseille, France; ^2^ Assistance Publique-Hôpitaux de Marseille (AP-HM), Hôpital de la Timone, Unité d’hépatologie, Marseille, France; ^3^ Assistance Publique-Hôpitaux de Marseille (AP-HM), Hôpital de la Timone, Laboratoire de Biochimie, Marseille, France; ^4^ C2VN, INSERM 1263, INRAE 1260, Team 5, Aix-Marseille Université, Marseille, France; ^5^ IHU Méditerranée Infection, Marseille, France

**Keywords:** non-alcoholic steato-hepatitis, metabolic-associated fatty liver disease, endogenous ethanol, gut microbiota, enterocloster bolteae, limosilactobacillus fermentum, mediterraneribacter gnavus, streptococcus mutans

## Abstract

**Background:**

Non-alcoholic steatohepatitis (NASH) has become a major public health issue as one of the leading causes of liver disease and transplantation worldwide. The instrumental role of the gut microbiota is emerging but still under investigation. Endogenous ethanol (EtOH) production by gut bacteria and yeasts is an emerging putative mechanism. Microbial metagenomics and culture studies targeting enterobacteria or yeasts have been reported, but no culturomics studies have been conducted so far.

**Aim:**

To assess fecal EtOH and other biochemical parameters, characterize NASH-associated dysbiosis and identify EtOH-producing gut microbes associated with the disease, fecal samples from 41 NASH patients and 24 controls were analyzed. High-performance liquid chromatography (HPLC) was used for EtOH, glucose, total proteins, triglyceride and total cholesterol. Viable bacteria were assessed with microbial culturomics. Microbial genetic material was assessed using 16S metagenomics targeting the hypervariable V3V4 region.

**Results:**

Fecal EtOH and glucose was elevated in the stools of NASH patients (p < 0.05) but not triglyceride, total cholesterol or proteins. In culturomics, EtOH-producing *Enterocloster bolteae* and *Limosilactobacillus fermentum* were enriched in NASH. V3V4 16S rRNA amplicon sequencing confirmed the enrichment in EtOH-producing bacteria including *L. fermentum*, *Mediterraneibacter gnavus* and *Streptococcus mutans*, species previously associated with NASH and other dysbiosis-associated diseases. Strikingly, *E. bolteae* was identified only by culturomics. The well-known *Lacticaseibacillus casei* was identified in controls but never isolated in patients with NASH (p < 0.05).

**Conclusion:**

Elevated fecal EtOH and glucose is a feature of NASH. Several different EtOH-producing gut bacteria may play an instrumental role in the disease. Culturomics and metagenomics, two complementary methods, will be critical to identify EtOH-producing bacteria for future diagnostic markers and therapeutic targets for NASH. Suppression of EtOH-producing gut microbes and *L. casei* administration are options to be tested in NASH treatment.

## Introduction

Non-alcoholic fatty liver disease (NAFLD), including its most severe form, non-alcoholic steatohepatitis (NASH), is becoming a major public health issue (Younossi et al., 2016). In a recent meta-analysis, the prevalence of NASH was estimated between 1.50% and 6.45% of the general population. Overall, mortality incidence rates ranged from 11.77 (range 7.10-19.53) for NAFLD to 25.56 (range 6.29-103.80) per 1,000 person-years for NASH (Younossi et al., 2016). According to the 2018 Global Burden of Disease study, the prevalence of NAFLD increases exponentially up to 25% worldwide alongside metabolic syndrome and diabetes (Younossi et al., 2018) and will soon be the leading cause of liver transplantation worldwide (Haldar et al., 2019). Although viral hepatitis remains the leading cause of mortality, NAFLD is the fastest-growing cause of liver morbidity and mortality (Paik et al., 2020). For example, age-standardized mortality rates are falling for HBV, HCV, and ALD (alcohol liver disease), but this rate is rising for NAFLD (Paik et al., 2020).

Dysbiosis of the gut microbiota has already been associated with metabolic diseases including diabetes (Wu et al., 2023), obesity (Million et al., 2012), dyslipidemia (Guo et al., 2022) but its involvement in NAFLD and more specifically NASH is not clearly established. Recent studies have shown using metagenomics a dysbiotic profile in NASH patients characterized by an increase in *Proteobacteria* at the phylum level (Zhu et al., 2013; Yuan et al., 2019), *Lactobacillaceae* (Meijnikman et al., 2022) and *Enterobacteriaceae* (Zhu et al., 2013; Yuan et al., 2019) at the family level, and *Lactobacillus*, *Clostridium* and *Escherichia* (Zhu et al., 2013; Yuan et al., 2019; Jin and Xu, 2023) at the genus level.

Endogenous ethanol (EtOH) production by gut microbes has recently (Zhu et al., 2013) been identified as one of the mechanisms underlying the role of the gut microbiota in the pathogenesis of NASH (Zhu et al., 2013; Yuan et al., 2019). Zhu et al. found high concentrations of EtOH in the blood of NASH patients, confirmed in patients’ stools by Yuan et al. (Zhu et al., 2013; Yuan et al., 2019). Case-control studies have shown the involvement of specific microorganisms in endogenous EtOH production, notably yeasts of the *Candida* and *Pichia* genus (Mbaye et al., 2022) and bacteria including *Klebsiella pneumoniae*, *Limosilactobacillus fermentum* and *Lactococcus lactis* (Mbaye et al., 2023; Yuan et al., 2019).

The *Clostridium* genus, which was previously associated with NASH (Zhu et al., 2013; Jin and Xu, 2023), has recently been reclassified into several new genera including the *Enterocloster* genus that consists of six species (Haas and Blanchard, 2020). *Enterocloster* spp. have already been associated with diseases. *Enterocloster citroniae* was associated with type 2 diabetes (Ruuskanen et al., 2022), *Enterocloster clostridioformis* with fatty liver (Ruuskanen et al., 2021) and *Enterocloster bolteae* with autism, diabetes, and fatty liver (Ruuskanen et al., 2021; Frame et al., 2023). Two species of this genus, *E. bolteae* and *Enterocloster asparagiformis*, are known to produce EtOH *in vitro* (Mohan et al., 2006).

To date, no culturomics studies have been carried out to characterize the microbiota of NASH patients. This untargeted omics method remains essential and complementary to metagenomics for exploring the microbiota. Indeed, this approach yields different but complementary results for gut microbial profiling. Indeed, it is a high throughput culture approach which is based on the multiplication of culture conditions with a variation of physico-chemical parameters such as culture media, atmosphere, and temperature, combined with MALDI-TOF MS for identification of the generated colonies. It thus allowed the isolation of over 300 previously unknown bacterial species in the human gut including species that were considered uncultivable using conventional culture methods (Lagier et al., 2016). This method is invaluable as only culture-based approaches provides living bacterial strains from complex samples (Lagier et al., 2012a; Tidjani Alou et al., 2017) which can be studied further and potentially be used as probiotics for targeted bacteriotherapy (Cao et al., 2023; Song et al., 2023).

In this study, we assessed biochemical parameters specially focusing on fecal EtOH concentration. Culturomics was used for the first time to characterize the composition of the gut microbiota of NASH patients compared with that of healthy controls. 16S rRNA targeted amplicon sequencing was used as a complementary method. Comparison between the two methods was performed, expected to yield different but overlapping results, as previously published (Lagier et al., 2012b).

## Methods

### Participants

We performed a case-control study comparing the fecal EtOH and microbiota using culturomics and metagenomics methods. Participants were recruited in the hepatology department where they were monitored. Cases were diagnosed with NASH according to recruitment criteria described in our previous study (Mbaye et al., 2022) whereas controls were individuals with no diagnosed liver disease. Stool collection occurred between January and June 2022. It is noteworthy that no blood samples were collected for this study. Exclusion criteria for both groups were antibiotics within the month prior to sampling, excessive alcohol consumption (men ≥ 30 g/d, women ≥ 20 g/d), steatosis-inducing drugs, and refusal of consent by the participant. Participants were not instructed to fast or abstain from drinking alcohol prior to submitting stool samples.

### Ethical considerations

This study was conducted upon obtaining the approval of the Local Ethics Committee and “Comité de Protection des Personnes” (CPP: 21.04391.000046 - 21075). The informed and written consent of each participant was collected in compliance with the Declaration of Helsinki (World Medical Association., 2013).

### Samples

As we previously showed that culturomics efficiently discriminates cases from controls in another nutritional disease (severe acute malnutrition (Pham et al., 2019)) and only highlights viable bacteria (Bellali et al., 2019), we primarily focused on culturomics results (14 NASH and 10 controls). Not all samples could be analyzed by culturomics as it is a time-consuming method compared to V3V4 16S rRNA amplicon sequencing (culturomics ~ 6 weeks per sample with ~ 5,000 colonies per sample, V3V4 16S rRNA sequencing ~ 1 week for 96 samples). Therefore, the number of samples analyzed using culturomics simultaneously was limited (a maximum of three samples due to the extensive workload) and we aimed at analyzing at least one control for one or two cases simultaneously. Thus, only the first 24 samples (14 NASH and 10 controls) were analyzed using culturomics whereas all samples (41 NASH and 24 controls) were analyzed using V3V4 16S amplicon sequencing simultaneously. No sample was common to our previous studies (Mbaye et al., 2022; Mbaye et al., 2023) allowing us to test reproducibility of our previous findings using different methods (specific yeast and enterobacterial microbial culture were used in our previous study but not untargeted culturomics). Characteristics of the population in this study are described in **Table 1**.

**Table 1:** Baseline characteristics.

	Controls (n = 24)	NASH (n = 41)
Demographic characteristics		
Age: mean ± SD	63.8 ± 7.6	64.2 ± 9.0
Female ratio (%)	14 (58%)	18 (44%)
Comorbidities		
Alcohol consumption	8 (33%)	5 (12%)
EtOH intake < 20 g/j	8 (33%)	2 (5%)
EtOH intake ≥ 20 g/j	0 (0%)	3 (7%)
Body Mass Index (kg/m^2^): mean ± SD	26.0± 2.5	30.0 ± 5.0
Normal weight (18.5 < BMI < 24.9)	12	4
Overweight (25.0 ≤ BMI < 29.9)	11	21
**Obesity**	1 (4%)	16 (39%)
Moderate obesity (30.0 ≤ BMI < 34.9)	1	10
Severe obesity (35.0 ≤ BMI < 39.9)	0	5
Morbid obesity (BMI ≥ 40)	0	1
Diabetes mellitus (%)	0 (0%)	24 (59%)
Hypertension (%)	4 (17%)	26 (63%)
Dyslipidemia (%)	0 (0%)	19 (46%)
Smoking (%)	0 (0%)	6 (15%)
Liver fibrosis stage		
F1	0 (0%)	6 (15%)
F2	0 (0%)	6 (15%)
F3	0 (0%)	8 (19%)
F4	0 (0%)	17 (41%)
No information on fibrosis stage	0 (0%)	1 (2%)
Hepatocellular carcinoma	0	8 (19%)
Ascitis	0	3 (7%)

BMI, Body mass index; NASH, Nonalcoholic steatohepatitis; SD, standard deviation.

### Measurement of fecal ethanol

For biochemical measurements, 0.05g of stool was suspended in 1.5 mL of high-performance liquid chromatography (HPLC) water in Eppendorf tubes. The tubes were homogenized and centrifuged at 250 rpm for 15 to 20 minutes. 1mL of the supernatant was collected after filtration at 0.22 µm in freeze-drying tubes to perform the measurement of biochemical parameters (EtOH, glucose, triglycerides, total proteins and cholesterol) using the Atellica® Solution Immunoassay and Clinical Chemistry Analyser (Siemens Healthineers, Saint-Denis, France) according to the manufacturer’s instructions.

### Bacterial isolation using culturomics

Microbial culturomics was used to explore the bacterial diversity of our stool samples generating a large number of colonies due to the various culture conditions used which identification is facilitated by the use of the MALDI-TOF MS technology as explained below.

For that purpose, direct inoculation of the stool sample was performed with 0.3 g of stool resuspended in 1 mL of 1x PBS. Ten serial dilutions of this suspension were realized, and 50 µL of each dilution was spread on 5% Columbia (COS) agar enriched with sheep blood (bioMerieux Marcy l’Etoile, France) and modified YCFA (yeast extract, casein hydrolysate, fatty acids) agar (https://www.dsmz.de/microorganisms/medium/pdf/DSMZ_Medium1611.pdf). Inoculated agar plates were incubated in aerobic and anaerobic atmospheres at 37°C for 48 hours.

Additionally, enrichments were made in liquid broths. Anaerobic culture conditions consisted of 200 µL of each sample inoculated in anaerobic blood culture bottles (bioMerieux, Durham, NC, USA) and YCFA medium, both supplemented with 5% of defibrinated sheep blood and 5% of 0.22 µm filtered rumen fluid. Serial dilutions of each culture were inoculated on COS agar and YCFA agar over a one-month period at different timepoints (24h, day 3, day 7, day 10, day 15, day 21, day 30). The colonies obtained were sub-cultured after a 48 hour-incubation at 37°C using Zip bag (Oxoid, Dardilly, France) containing an anaerobic generator, gasPak (Becton Dickinson, Le Pont de Claix, France). An aerobic condition was also performed with 200 µL of stool inoculated into an aerobic blood culture bottle (BioMerieux, Marcy l’Etoile, France) medium supplemented with 5% of defibrinated sheep blood and 5% of 0.22 µm filtered rumen fluid. Serial dilutions were inoculated on COS agar over a one-month period at different timepoints (24h, day 3, day 7, day 10, day 15, day 21, day 30) and incubated for 24 hours at 37°C in an aerobic atmosphere. Colonies were sub-cultured in the same conditions. Purified colonies were identified using MALDI-TOF MS and genome sequencing when necessary (Lagier et al., 2012a). Briefly, isolated colonies were then plated on a 96 MSP plate with 2ul of pre-prepared matrix solution containing saturated α-cyano-4-hydroxycinnamic acid, 50% acetonitrile and 2.5% trifluoroacetic acid. Spectra were recorded in positive linear mode. Data were automatically acquired using FlexControl v.3.4 and MALDI Biotyper Compass v4.1 software for assay preparation and biotyping analysis. Spectra from different species were compared with the MBT Compass BDAL v.11 library (Bruker) containing 10,833 spectra references (3,893 species), as well as with our culturomics laboratory database containing 9,973 spectra references (2,186 bacterial species). Identification at the species level was obtained for a spectrum matched with a score higher than 1.9. Colonies unidentified using MALDI-TOF MS(score < 1.9) were identified using genome sequencing (Fournier et al., 2015).

### V3V4 16S rRNA amplicon sequencing

#### DNA extraction was realised with two different protocol

Method 1 DNA was extracted using the E.Z.N.A Tissue DNA kit (omega BIO-TEK, 400 Pinnacle Way suite 450, Norcross, GA 30071, USA). Method 2 fecal oeses were put into a tube that contains 500μL of PBS, glass powder and glass beads, to perform mechanical lysis using fastprep (90 seconds, 6.5m/sec). After decanting for 1min, the supernatant is collected. The latter was then centrifuged at 12000rpm for one minute and the supernatant was eliminated. On the base, 19μL of mixture containing 2μL glycoprotein denaturation buffer 10x (New England Biolabs) and 17μL H2O were added to denature, heat to 100°C for 10 min.

Deglycolization of the samples was achieved by adding a mixture containing 2μL of reaction buffer G3 10X (New England Biolabs), 2μL of EndoHf (New England Biolabs), 2μL of cellulase (SIGMA, France) and 16μL H2O Incubate at 37°C overnight. The next day, 25 μL of proteinase K (Euromedex, 24 Rue des Tuileries, 67460 Souffelweyersheim) and 180μL of buffer G2 (Qiagen, Hilden, Germany) were added to the tubes. After incubation for 1 hour at 56°C and centrifugation for 1min at 1100g, 200μL of supernatant was collected in an EZ1 flat-bottomed tube for the EZ1 tissue protocol from Qiagen (Hilden, Germany).

For each extraction protocol, genomic DNA was amplified for the 16S “V3-V4” regions by PCR for 45 cycles, using the Kapa HiFi Hotstart ReadyMix (Kapa Biosystems Inc,Wilmington, MAU.S.A), we use V3_V4 primers with adapters (FwOvAd_341FTCGTCGGCAGCGTCAGATGTGTATAAGAGACAGCCTACGGGNGGCWGCAG; RevOvAd_785RGTCTCGTGGGCTCGGAGATGTGTATAAGAGACAGGACTACHVGGGTATCTAATCC). After purification on CleanNGS (CleanNA Coenecoop 75 2741 PH Waddinxveen, the Netherlands), the concentration was measured with High sensitivity Qubit technology (Life technologies, Carlsbad, CA, USA). Library of each extraction method were dilute at 3,5 ng/uL and pooled (1:1). Illumina sequencing adapters and dual-index barcodes were added to amplicons by pcr. After purification on CleanNGS (CleanNA

Coenecoop 75 2741 PH Waddinxveen, the Netherlands), this library was pooled with others multiplexed samples. The global concentration was quantified by a Qubit with the high sensitivity kit (Life technologies, Carlsbad, CA, USA). Pooled amplicons were dilute to obtain a 8 pM library and we added 15% of PhiX control. The 16S rRNA were sequenced on MiSeq technology (Illumina, Inc, San Diego CA 92121, USA) on 500 cycle cartridges. Automated cluster generation and paired- end sequencing with dual index reads were performed in single 39-hours run in a 2x250bp. The paired reads were filtered according to the read qualities. Taxonomic assignment was performed using the MetaGX pipeline (developed in our center) as previously described (Bellali et al., 2021).

### Comparison of culturomics and V3V4 16S amplicon sequencing results

To understand why some species were found to be associated with NASH in culture but not in V3V4 16S amplicon sequencing, we used blastN to match the complete reference sequence of the 16S rRNA gene of each species significantly associated with NASH (or absence of NASH) with the assembled 16S amplicon sequencing datasets prior to the initial taxonomical assignment. The reference sequence of each species was found using LPSN (https://www.bacterio.net/, last accessed October 23^rd^, 2023). BLASTn was applied to the 65 samples with the following thresholds: 97% identity and 95% coverage.

### Statistical analysis

To compare quantitative variables such as fecal EtOH concentration, the bilateral Mann-Whitney test was performed with GraphPad Prism version 9 (GraphPad Software, San Diego, CA, USA). For fecal ethanol and glucose, visual examination clearly showed that several samples exceeded the maximum value of healthy controls (upper normal value). This prompted us to check whether the proportion of samples with abnormal values could be due to chance. Barnard’s two-tailed exact test was used. A p-value < 0.05 was considered significant. To compare the microbiota profile obtained by culturomics, the detection frequency difference of each species was calculated (as relative abundance is not assessed by this technique). For that purpose, the occurrence of each species in both groups was estimated based on its presence/absence in each sample. Once the frequency of each species in each group was determined, the difference was calculated to determine which species are enriched in each group. The bilateral Chi-squared test was used to compare frequency differences. To compare the microbiota profile obtained by illumina MiSeq V3V4 16S gene sequencing, we used three statistical approaches: 1) frequency comparison within each group, which takes into account only the presence or absence of each species and most closely resembles culturomics, 2) linear discriminant analysis using the LefSE pipeline the Lefse pipeline (Afgan et al., 2018) in Galaxy (https://huttenhower.sph.harvard.edu/galaxy/) and finally, and 3) linear discriminant analysis using the Microbiome analyst pipeline (Chong et al., 2020) (https://www.microbiomeanalyst.ca/). Alpha and beta-diversity were also assessed with this last pipeline.

## Results

### Participants

A total of 65 individuals were recruited for this study including 41 NASH patients and 24 healthy controls. For NASH patients, the mean age was 64.2 ± 9.0 years with a sex ratio of 1.3 (23/18 (M/F)), and the mean body mass index was 29.5 ± 5.0. Among the NASH patients, 9.75% had a normal weight, 51.2% were overweight, 24.4% were moderately obese, 12.2% were severely obese and 2.4% were morbidly obese. Among the NASH patients, 53.7% were diabetic and 28.8% had high blood pressure. For healthy controls, the mean age was 63.8 ± 7.6 years with a sex ratio of 10/14 (M/F), and the mean body mass index was 25.2 ± 2.5.

### Increased fecal ethanol in NASH

**Figure 1:**
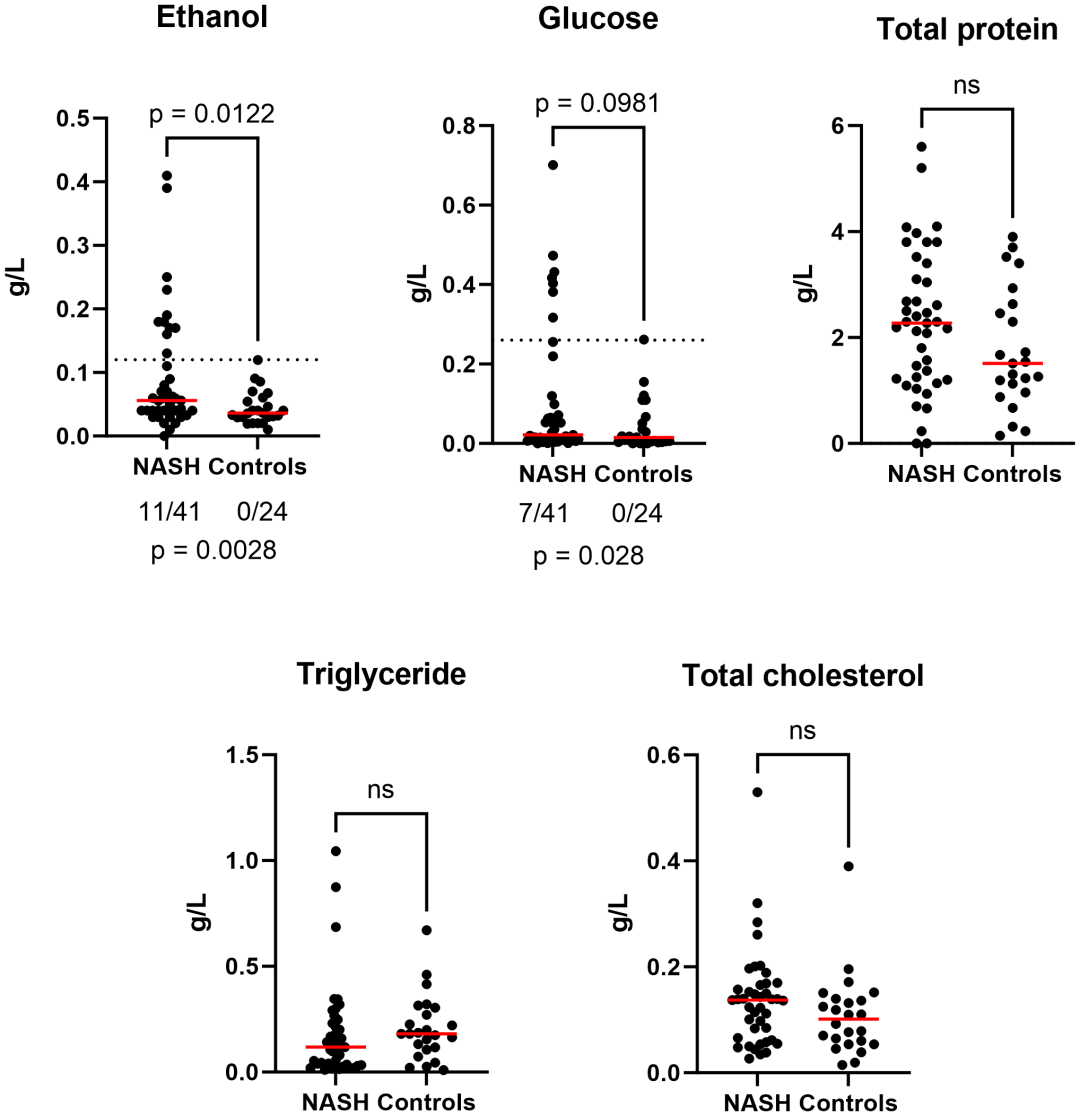
Anormal increased fecal ethanol and glucose concentration in NASH (n = 65). Bilateral Mann-Whitney test was used to estimate significance for all graphs (ns : not significant). Additionally, for fecal ethanol and glucose, we evaluated if the proportion of anormal values was due to chance using bilateral Barnard’s test (see p-value below). Raw data provided in **Supplementary Table 1**.

The fecal EtOH concentration was significantly higher in the stools of NASH patients compared to healthy controls (p-value= 0.0145, **Figure 1**). By setting a threshold at 0.12 g/L (maximal value of healthy controls), 11/41 NASH presented abnormal values (vs 0/24, p = 0.0028). There was a trend for increased fecal glucose concentration (p = 0.098) with 7/41 abnormal values (vs 0/24, p = 0.028). In contrast, none of the other biochemical parameters were significantly different between the two groups (**Figure 1**; **Supplementary Table 1**).

### Culturomics results

**Figure 2:**
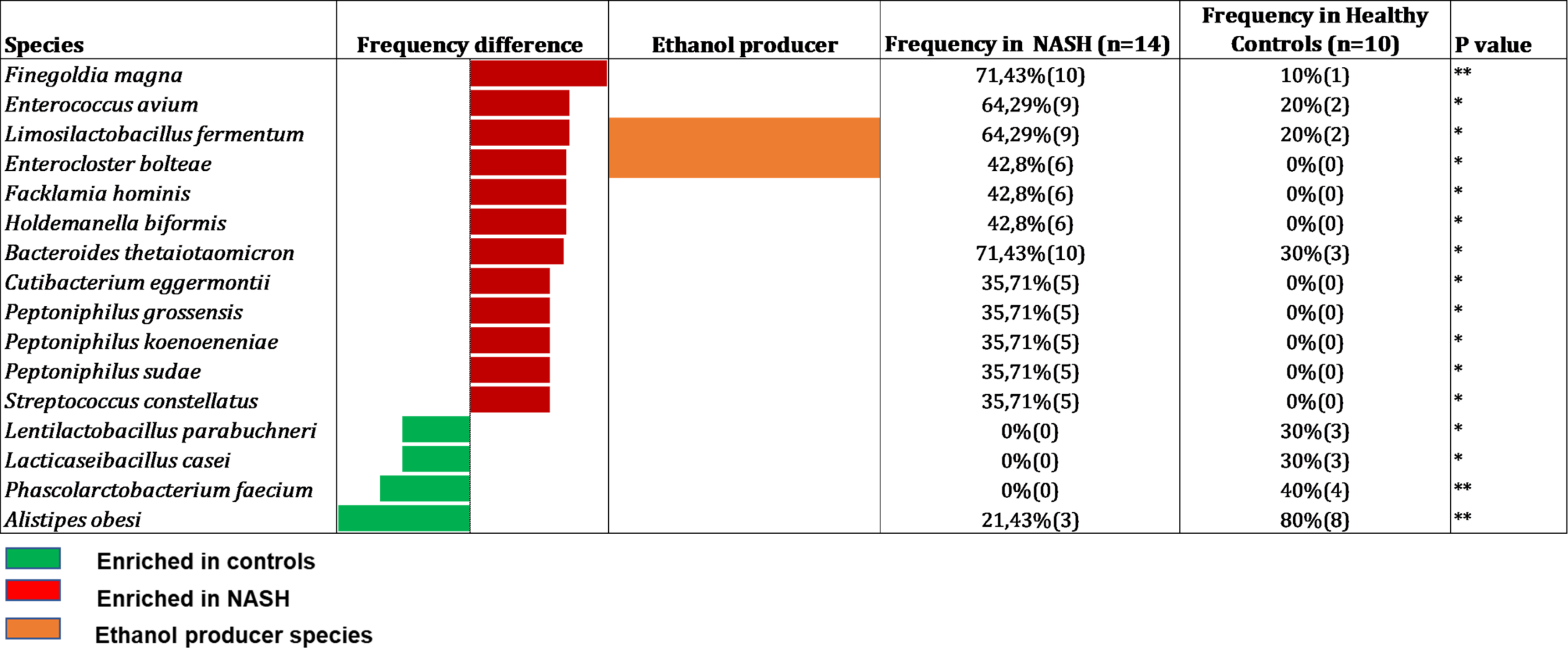
Species with a significant frequency difference between NASH and Healthy controls in Culturomics (n = 24). In red species enriched in NASH, in green species enriched in controls and in orange: species capable to produce ethanol according to the literature. Raw data provided in **Supplementary Table 2**.

Culturomics was performed on 24 samples (14 NASH and 10 controls). A total of 34,560 colonies (mean 1,440 colonies per sample) were isolated allowing to identify 358 different bacterial species. These bacterial species were distributed across 11 phyla and 143 genera. 168 species were common to both groups (NASH and controls) whereas 57 species were specific to the control group and 163 to the NASH group (**Supplementary Table 2**). Among the 358 identified species, only 16 (4.5%) were associated with a significant detection frequency difference including 12 significantly more frequent in the NASH group and 4 more frequent in the control group (**Figure 2**). As we focused on EtOH-producing microbes, we observed that, among the 12 species detected more frequently in the NASH group, two were known to produce EtOH: *Limosilactobacillus fermentum* and *Enterocloster bolteae.* Moreover, eight increased species in NASH patients were specific to the group (not detected in 10 controls) with the most represented being *Enterocloster bolteae*, *Flacklamia hominis* and *Holdemanella biformis* (all detected in 6/14 (42.9%) of NASH patients vs 0/10 (0%) controls, p=0.013). Similarly, among the four significantly increased species in controls, three species were specific to the group (not detected in the 14 cases, **Figure 2**), two of which, *Lactilactobacillus casei* and *Phascolarctobacterium feacium*, were known to be consistently associated with health (Wu et al., 2017; Martens et al., 2022). Interestingly, *Alistipes obesi* (renamed *Alistipes communis*), a species previously discovered using the culturomics approach in our center (Hugon et al., 2013), was the most enriched in controls (3/14 vs 8/10, p= 0.006) and has recently been associated with health (Yuan et al., 2023).

### Culturomics identified six new species

Unlike amplicon sequencing, culturomics has the unequaled advantage to isolate hitherto unknown species. In this study, we isolated six new species, including 2 from new genera, the description of which is ongoing. For these species, two were isolated from two different NASH patients (*Lancefieldella massiliensis* sp. nov. and *Faecibacter massiliensis* gen. nov. sp. nov.) and four from a single control sample (*Lachnospira massiliensis* sp. nov., *Ligilactobacillus massiliensis* sp. nov.*, Phamibacteria massiliensis* gen. nov. sp. nov. and *Pusillibacter massiliensis* sp. nov.). However, these species were not discriminant in both groups.

### V3V4 16S rRNA amplicon sequencing results

After usual Illumina sequencing targeting the V3V4 region of the 16S rRNA gene (**Supplementary Table 3**, bioproject number PRJEB62828), the diversity was assessed using the Microbiome analyst pipeline (Chong et al., 2020). Alpha-diversity Simpson index was significantly lower in NASH patients. Strikingly, 7 NASH patients had very low Simpson indexes (**Supplementary Figure 1a**). Beta-diversity evidenced an increased heterogeneity of NASH samples compared to controls (**Supplementary Figure 1b**). Dendrogram of microbiota profile at the species level did not show a clear distinction between cases and controls (**Supplementary Figure 1c**). This prompted us to focused on species with a significant difference of frequency or abundance between the two groups.

Using the Microbiome analyst pipeline (Chong et al., 2020) again, a linear discriminant analysis (LDA) between the two groups (41 NASH and 24 controls) was performed at different taxonomic levels (phylum, family, genus, and species). At the phylum level, the LDA showed a significant increase of *Candidatus* Saccharibacteria (a superphylum belonging to the Candidate Phyla Radiation (CPR) division) and *Actinobacteria* in NASH patients (**Supplementary Figure 2a**). At the family level, we found an enrichment in *Streptococcaceae*, *Coriobacteriaceae* and *Lachnospiraceae* (**Supplementary Figure 2b**). Consistently, the genera *Streptococcus* (*Streptococcaceae*) and *Blautia* (*Lachnospiraceae*) were also increased (**Supplementary Figure 2c**).

Conversely, the phyla *Bacteroidetes* and *Euryarchaeota* were decreased in cases whereas at the family level a decrease of *Bacteroidaceae*, *Methanobacteriaceae*, *Rikenellaceae* and *Eubacteriaceae* was observed. At the genus level, *Bacteroides*, *Methanobrevibacter*, *Alistipes*, *Paraoscillibacter*, *Romboutsia* and *Phascolarctobacterium* were associated with controls (**Figure S2a, b and c**).

**Figure 3:**
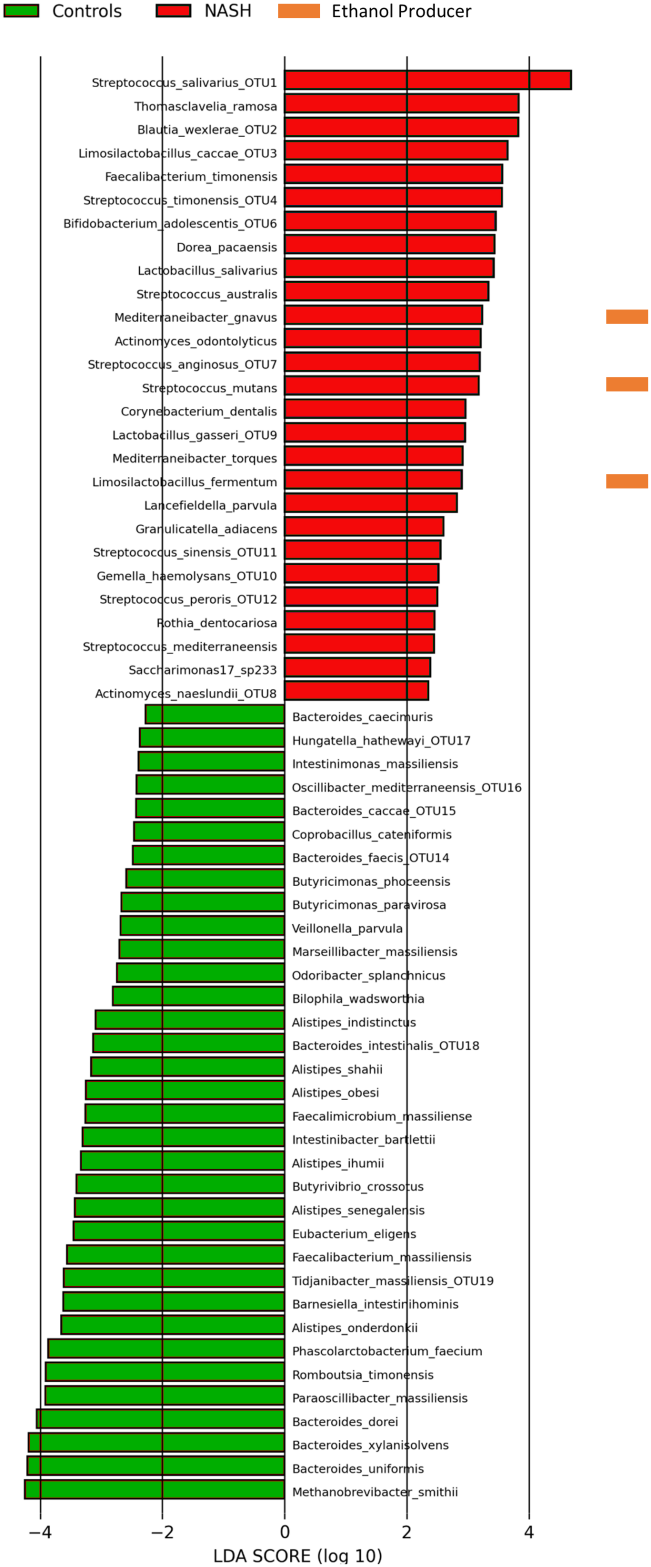
Species associated with NASH or controls by Linear discriminant analysis (16S metagenomics, n = 65). Linear discriminant analysis performed using the Galaxy Hutlab (https://huttenhower.sph.harvard.edu/galaxy/) with default parameters. Ethanol producers identified by literature search. Species with an OTU number corresponded to OTUs assigned to multiple species (see **Supplementary Table 4**). Other OTUs were assigned to only one species. Saccharimonas12_sp233 corresponded to Candidatus *Nanogingivalacae*, a member of the Candidate Phylum Radiation (see text). Raw data provided in **Supplementary Table 3**.

At the species level, we primarily used the LefSE pipeline (Afgan et al., 2018) to perform the linear discriminant analysis (**Figure 3**) but also the Microbiome analyst (Chong et al., 2020) at the species level (**Figure S3**). The NASH group was associated with *Streptococcus* spp., *Blautia* spp., *Thomasclavelia ramosa* (formerly *Clostridium ramosum* then *Erysipelatoclostridium ramosum)*, *Limosilactobacillus fermentum* and *Faecalibacterium timonensis* (**Supplementary Figure 3**). Healthy controls presented an increased abundance of species, previously associated with health in the literature, namely *Methanobrevibacter smithii*, *Bacteroides* spp. (8 OTUs corresponding to at least 8 *Bacteroides* species, *Romboutsia timonensis* and *Phascolarcobacterium faecium*, **Figure 3**). Interestingly, the syntrophic taxa, *Methanobrevibacter* spp. and *Bacteroides* spp. were consistently associated with controls in our study, at all taxonomic levels (Samuel and Gordon, 2006; Djemai et al., 2022). The consistency of the depletion of the association between *Methanobrevibacter smithii* and *Bacteroides* species is consistent with the loss of the archaeal-bacterial mutualism reported several years ago (Samuel and Gordon, 2006). Finally, only the LefSE pipeline allow us to identify 3 EtOH-producing species, all enriched in NASH, namely *Mediterraneibacter gnavus* (formerly *Ruminococcus gnavus*), *Streptococcus mutans* and *Limosilactobacillus fermentum* (**Figure 3**).

### Comparison of culturomics V3V4 16S amplicon sequencing results

The data obtained using culturomics and 16S amplicon sequencing were analyzed using two different analyses: detection frequency difference (which considers the presence/absence of the species in the sample) performed the culturomics and amplicon sequencing data and LDA (linear discriminant analysis, which considers the relative abundance of species) performed only for the amplicon sequencing datasets. By comparing these two methods, only three species were common for culturomics and 16S metagenomics: the EtOH-producing *Limosilactobacillus fermentum* enriched in NASH patients whereas two species were enriched in controls namely *Phascolarctobacterium faecium* and *Alistipes obesi* (rename *Alistipes communis*) in controls (**Figure 2** & **3** and **Figure S5**).

Interestingly, lactic acid bacteria represented 48% (12/25) of species enriched in NASH patients, the most represented (present in more than 50% of patients) being *Streptococcus salivarius* (40/41), *Streptoccocus anginosus* (25/41) and *Streptococcus australis* (23/41). Species enriched in NASH also included *Mediterraneibacter* [*Ruminococcus*] *gnavus*, *Thomasclavelia ramosa*, *Gemella haemolysans*, *Faecalibacterium timonensis* and *Granulicatella adiacens* (**Figure 3**). It is noteworthy that among the species enriched in the NASH group, seven were totally absent in controls: *Actinomyces naeslundii*, *Bifidobacterium adolescentis*, *Limosilactobacillus fermentum*, *Limosilactobacillus caccae, Lactobacillus salivarius*, *Streptococcus sinensis* and an unknown member of the Candidate Phyla Radiation (CPR, called saccharimonas17 sp233) classified with a new unclassified family *Candidatus* Nanogingivalaceae (**Figure 3**).

Conversely, 36 species were found to have strong associations with controls with a predominance of the *Bacteroides* genus (23 species) and health-associated species previously found with the LDA and/or culturomics: *P. faecium*, *R. timonensis* and *A. obesi* (**Figure 3**). These species also included the main human gut archaeal *M. smithii*, as well as several members of the *Bacteroides* genus for which syntrophic interactions are well described.

### Four EtOH-producing bacteria associated with NASH identified by culturomics and 16S metagenomics

Endogenous EtOH production is an emerging mechanism described in the pathophysiology of NASH (Zhu et al., 2013; Yuan et al., 2019; Mbaye et al., 2022; Mbaye et al., 2023). Among all the species with a significant difference between NASH patients and controls, only four were known to produce EtOH and 4/4 (100%) were enriched in NASH patients (binomial test, p = 0.025) by culture and/or sequencing (**Table 2**; **Figure 2**, **3**). In contrast, none of the species enriched in the controls were known to produce EtOH (**Figure 3**). These four species included a member of the recently reclassified *Limosilactobacillus* genus (Zheng et al., 2020), *L. fermentum*, a EtOH producer according to the literature (Elshaghabee et al., 2016), was found enriched in both culture and sequencing and has previously been associated with EtOH production in NASH (Meijnikman et al., 2022; Mbaye et al., 2023) (**Table 2**). In addition, *S. mutans*, a species of the genus *Streptococcus* able to produce EtOH (Takahashi et al., 1991), was associated with NASH in the amplicon sequencing datasets. The *Streptococcus* genus was previously associated with NASH (Naka et al., 2014) and includes *S. mutans*, a known cariogenic species (Alaluusua and Renkonen, 1983). The other two EtOH-producing species, *E. bolteae* (which ethanol production has been shown *in vitro* with strains enriched in hepatitis B patients (Magdy Wasfy et al., 2023)) and *Mediterraneibacter gnavus* (Hynönen et al., 2016), were found to be associated with NASH for the first time in this study. Specifically, *E. bolteae* was found enriched in NASH patients only using the culturomics approach. This species was recently associated with autism and hepatic steatosis (Ruuskanen et al., 2021; Frame et al., 2023). *Mediterraneibacter gnavus*, reclassified by Togo et al. (Togo et al., 2018), has been associated with several pathologies including obesity (Chen et al., 2021; Lin et al., 2023),, intestinal inflammation (Hall et al., 2017), allergies in children (Chua et al., 2018) and non-alcoholic steatosis (Hullar et al., 2021). These four species (**Table 2**) should be added to the repertoire of gut microbes possibly instrumental in NASH through endogenous ethanol production. As *E. bolteae* was only found using culture-based approaches as well as other species of interest (**Table S5**) and the V3V4 region of the 16S rRNA gene does not consistently allow unambiguous taxonomic assignment of OTUs, we conducted a BLASTn matching the complete reference sequence of the 16S rRNA gene (**Table S4**) of the 15 species of interest highlighted using culturomics against the 16S amplicon datasets (**Figure S4**, **Table S5**). This analysis showed that *E. bolteae* was also detected using 16S amplicon sequencing although it was depleted in NASH patients whereas *L. fermentum* was also found enriched in NASH patients.

**Table 2:** Four EtOH-producing species found enriched in NASH according to microbiological and statistical methods.

	Culturomics	V3V4 16S amplicon sequencing			
	N = 24	Step 1 (n = 24)		Step 2 (n = 65)	
	Frequency difference	Frequency difference	Linear discriminant analysis	Frequency difference	Linear discriminant analysis
*Limosilactobacillus fermentum* **^EtOH^**	**X**	**X**	**X**	**X**	**X**
*Mediterraneibacter_gnavus* **^EtOH^**				**X**	**X**
*Streptococcus_mutans* **^EtOH^**				**X**	**X**
*Enterocloster bolteae* **^EtOH^**	**X**				

Table comparing technique (culturomics & 16S metagenomics) or statistical (frequency difference or linear discriminant analysis) methods, and according to samples (24 analyzed both by culturomics and metagenomics (Step 1) and 41 analyzed only by 16S metagenomics). Step 2 included all samples. *L. fermentum* was the only species found by culturomics and 16S metagenomics, whatever the group of samples included (24 analyzed both by culturomics and metagenomics or 65 analyzed by 16S metagenomics). *Enterocloster bolteae* was identified only by culturomics. Its instrumental role is highly suspected based on several reports associating this species with dysbiosis related diseases (see Discussion). These four species should be added to the repertoire of gut microbes possibly instrumental in the disease through endogenous ethanol production.

## Discussion

In this study, we confirmed the intestinal dysbiosis associated with NASH using culture-based as well as culture-independent approaches. We first showed an increase in fecal EtOH concentration in NASH patients, confirming results already described in the literature. The NASH-associated dysbiosis found in this study confirms those obtained in previous studies. It was characterized by an increase of *Streptococcaceae* (Vernekar et al., 2018; Meijnikman et al., 2022) and *Lachnospiraceae* at the family level and *Streptoccocus*, *Limosilactobacillus* and *Blautia* at the genus level (Abdugheni et al., 2022; Meijnikman et al., 2022). Interestingly, EtOH-producing species (*E. bolteae, M. gnavus, L. fermentum*, and *S. mutans*) were enriched in NASH patients. The association of *T. ramosa* and *L. fermentum* with NASH confirms our previous findings (with different patients) and other reports in the literature (Loomba et al., 2019; Meijnikman et al., 2022; Mbaye et al, 2023).

Our study evidence a plausible association of *E. bolteae* with NASH through endogenous EtOH production. The bacterium was first isolated from autistic patients (Song et al., 2003) and subsequently repeatedly associated with this disease (Cox et al., 2021; Pandit et al., 2021; Frame et al., 2023). It is strict anaerobic, gram-positive, rod-shaped and measures 1.0-1.2 µm in diameter. Acetate and lactate are the main end products of glucose metabolism. Other sugars metabolized by the bacterium include arabinose, fructose, sucrose, glycerol, maltose, mannose, melezitose, sorbitol, trehalose and xylose. It is sensitive to kanamycin and colistin sulfate; intermediate to bacitracin, cefoxitin and clindamycin; and resistant to ampicillin, piperacillin, ticarcillin and vancomycin (Song et al., 2003). Its versatility to consume multiple sugars knowing that it produces ethanol makes it a suitable pathogen candidate. Indeed, a very frequent endogenous production of ethanol can be expected in humans who carry this bacterium even with varied sugar diets.

A 2019 metagenomics study found that this species was the best for classifying NAFLD according to the stage of fibrosis (Loomba et al., 2019). Moreover, *E. bolteae* has already been associated with intestinal dysbiosis in autistic patients (Frame et al., 2023) and chronic inflammatory bowel disease (Chen et al., 2023). This species, found only in the culture-based analysis in this study, is an EtOH producer. Endogenous EtOH production is potentially involved in the pathophysiology of the disease (Zhu et al., 2013; Yuan et al., 2019). *E. bolteae* was the only species found by culture and not by V3V4 16S rRNA amplicon sequencing. The lack of detection of *E. bolteae* in V3V4 16S amplicon sequencing might be due to the fact that these hypervariable regions of the 16S rRNA gene cannot discriminate species of the *Enterocloster* genus. In fact, this genus was recently created subsequently to multiple reclassifications of the *Clostridium* genus (Song et al., 2003). In fact, all OTUs classified within this genus were multi-assigned in the metadata (see **Supplementary table S3**). However, culturomics allowed the unambiguous identification of *Enterocloster* species: *Enterocloster bolteae* (6 in NASH vs. 0 in controls), *Enterocloster aldenensis* (3 in NASH vs. 0 in controls), *Enterocloster citroniae* (2 in NASH vs. 1 in controls), *Enterocloster clostridioformis* (2 in NASH vs. 0 in controls) and *Enterocloster lavalensis* (1 in NASH vs. 0 in controls). According to the microbial culturomics (Lagier et al., 2012a) approach, the media used for culture were not specific for any specific microbial population, but rather mimicked the sample environment to explore the bacterial population of the sample as exhaustively as possible. For instance, YCFA medium, rumen and anaerobic atmospheres were used to reproduce the physiologic environment (Tidjani Alou et al., 2020). This suggests the complementarity between these methods to explore gut microbiome (Lagier et al., 2018).

Future studies are needed to understand why the association between *E. bolteae* and NASH was detected using culturomics and shotgun metagenomic, and not 16S amplicon sequencing. Future experimental studies and *in vitro* tests will improve its instrumental role in the pathophysiology of NASH. Our results suggest the potential association of endogenous EtOH production by digestive microorganisms and NASH, and the use of new therapeutic avenues such as antibiotics (*E. bolteae* is expected to be sensitive to rifaximin which is effective in NASH in some studies (Abdel-Razik et al., 2018 2018; Boehme et al., 2023 2023), fecal transplantation or the use of phage therapy targeting *E. bolteae* as described by Yuan et al. with *Klebsiella pneumoniae* (Gan et al., 2023). Future studies will determine ethanol production by the 12 species (81 strains) enriched in NASH patients. In addition, the pathogenic role of these species in the physiopathology of NASH could explored in a murine model (Yuan et al., 2019).

In this study, we showed frequency difference did not represent the most relevant parameter that is predictive value. Indeed, even if rare, a microbial species is of particular interest if it is always associated with a disease or always associated with absence of the disease. Even if sample size was quite low due to microbial culturomics fastidious process, we were struck by the fact that the EtOH producing *E. bolteae* had a 100% positive predictive value, in contrast the probiotic *L. casei* has a 100% negative predictive value, suggesting that *E. bolteae* is a pathobiont (Lagier et al., 2018; Abdel-Razik et al., 2018 2018) and *L. casei* a probiotic potentially protective against NASH (Lee et al., 2020). And the probiotic effect of *L. casei* is known for around a century. Strikingly, this species was shown to interfere with ethanol production in co-culture with yeast (Spanhaak et al., 1998). Future studies are strongly needed to test if *L. casei* may have a beneficial effect on NASH.

One of the limitations of this study may be linked to the discrepancy in the number of samples analyzed using culturomics (24 samples) and 16S amplicon sequencing (65 samples) due to the extensive workload associated with the culturomics method.

Testing antimicrobial therapies for NASH without identifying instrumental gut microbes that might include both yeast (Demir et al., 2022; Mbaye et al., 2022) and bacteria is expecting to be unsuccessful. Current and previous studies from our center (Mbaye et al., 2022; Mbaye et al., 2023) and others strongly suggest that accurate profiling of the individual gut microbiota using both culturomics and metagenomics for yeasts, bacteria and archaea is likely to be a critical step for precision medicine using microbe-targeted therapies for NASH management in the future.

## Data availability statement

The datasets presented in this study can be found in online repositories. The names of the repository/repositories and accession number(s) can be found in the article/**Supplementary Material**.

## Ethics statement

This study involving humans was approved by the ethics committee of the IHU Méditerranée Infection under number 2020-004 and the “Comité de Protection des Personnes” under number CPP: 21.04391.000046 - 21075. The studies were conducted in accordance with the local legislation and institutional requirements. Written informed consent for participation in this study was provided by the participants’ legal guardians/next of kin. Written informed consent was obtained from the individual(s), and minor(s)’ legal guardian/next of kin, for the publication of any potentially identifiable images or data included in this article.

## Author contributions

BM: Formal analysis, Visualization, Writing – original draft, Investigation. RMW: Investigation, Writing – review & editing. PB: Resources, Writing – review & editing. MTA: Writing – review & editing. GM: Writing – review & editing, Data curation, Investigation. VB: Investigation, Data curation, Writing – review & editing. AC: Investigation, Data curation, Writing – review & editing. RG: Investigation, Resources, Writing – review & editing, Project administration, Supervision, Validation. MM: Conceptualization, Project administration, Validation, Formal analysis, Methodology, Supervision, Visualization, Writing – original draft, Writing – review & editing, Funding acquisition.

## References

[B1] Abdel-Razik A. Mousa N. Shabana W. Refaey M. Elzehery R. Elhelaly R.. (2018). Rifaximin in nonalcoholic fatty liver disease: hit multiple targets with a single shot. Eur. J. Gastroenterol. Hepatol. 30 (10), 1237–1246. doi: 10.1097/MEG.0000000000001232 30096092

[B2] Abdugheni R. Wang W-Z. Wang Y-J. Du M-X. Liu F-L. Zhou N.. (2022). Metabolite profiling of human-originated Lachnospiraceae at the strain level. iMeta 1 (4), e58. doi: 10.1002/imt2.58 PMC10989990 38867908

[B3] Afgan E. Baker D. Batut B. van den Beek M. Bouvier D. Cech M.. (2018). The Galaxy platform for accessible, reproducible and collaborative biomedical analyses: 2018 update. Nucleic Acids Res. 46 (W1), W537−44. doi: 10.1093/nar/gky379 29790989 PMC6030816

[B4] Alaluusua S. Renkonen O. V. (1983). *Streptococcus mutans* establishment and dental caries experience in children from 2 to 4 years old. Scand. J. Dent. Res. 91 (6), 453–457. doi: 10.1111/j.1600-0722.1983.tb00845.x 6581521

[B5] Bellali S. Lagier J-C. Million M. Anani H. Haddad G. Francis R.. (2021). Running after ghosts: are dead bacteria the dark matter of the human gut microbiota? Gut Microbes 13 (1), 1−12. doi: 10.1080/19490976.2021.1897208 PMC7993147 33757378

[B6] Bellali S. Lagier J. C. Raoult D. Bou Khalil J. (2019). Among live and dead bacteria, the optimization of sample collection and processing remains essential in recovering gut microbiota components. Front. Microbiol. 10. doi: 10.3389/fmicb.2019.01606 PMC6635563 31354688

[B7] Boehme M. Guzzetta K. E. Wasén C. Cox L. M. (2023). The gut microbiota is an emerging target for improving brain health during ageing. Gut microbiome 4, e2,1–e229. doi: 10.1017/gmb.2022.11 37179659 PMC10174391

[B8] Cao C. Shi M. Wang X. Yao Y. Zeng R. (2023). Effects of probiotics on non-alcoholic fatty liver disease: a review of human clinical trials. Front. Nutr. 10. doi: 10.3389/fnut.2023.1155306 PMC10349203 37457967

[B9] Chen H. Ou R. Tang N. Su W. Yang R. Yu X.. (2023). Alternation of the gut microbiota in irritable bowel syndrome: an integrated analysis based on multicenter amplicon sequencing data. J. Transl. Med. 21 (1), 117. doi: 10.1186/s12967-023-03953-7 36774467 PMC9921069

[B10] Chen X. Zhang D. Sun H. Jiang F. Shen Y. Wei P.. (2021). Characterization of the gut microbiota in Chinese children with overweight and obesity using 16S rRNA gene sequencing. PeerJ 9, e11439. doi: 10.7717/peerj.11439 34164233 PMC8194416

[B11] Chong J. Liu P. Zhou G. Xia J. (2020). Using Microbiome Analyst for comprehensive statistical, functional, and meta-analysis of microbiome data. Nat. Protoc. 15 (3), 799−821. doi: 10.1038/s41596-019-0264-1 31942082

[B12] Chua H-H. Chou H-C. Tung Y-L. Chiang B-L. Liao C-C. Liu H-H.. (2018). Intestinal dysbiosis featuring abundance of *ruminococcus gnavus* associates with allergic diseases in infants. Gastroenterology 154 (1), 154–167. doi: 10.1053/j.gastro.2017.09.006 28912020

[B13] Cox L. M. Maghzi A. H. Liu S. Tankou S. K. Dhang F. H. Willocq V.. (2021). Gut microbiome in progressive multiple sclerosis. Ann. Neurol. 89 (6), 1195–1211. doi: 10.1002/ana.26084 33876477 PMC8132291

[B14] Demir M. Lang S. Hartmann P. Duan Y. Martin A. Miyamoto Y.. (2022). The fecal mycobiome in non-alcoholic fatty liver disease. J. Hepatol. 76 (4), 788–799. doi: 10.1016/j.jhep.2021.11.029 34896404 PMC8981795

[B15] Djemai K. Drancourt M. Tidjani Alou M. (2022). Bacteria and methanogens in the human microbiome: a review of syntrophic interactions. Microb. Ecol. 83 (3), 536–554. doi: 10.1007/s00248-021-01796-7 34169332

[B16] Elshaghabee F. M. F. Bockelmann W. Meske D. de Vrese M. Walte H.-G. Schrezenmeir J.. (2016). Ethanol production by selected intestinal microorganisms and lactic acid bacteria growing under different nutritional conditions. Front. Microbiol. 7, 47. doi: 10.3389/fmicb.2016.00047 26858714 PMC4732544

[B17] Fournier P. E. Lagier J. C. Dubourg G. Raoult D. (2015). From culturomics to taxonomogenomics: A need to change the taxonomy of prokaryotes in clinical microbiology. Anaerobe 36, 73–78. doi: 10.1016/j.anaerobe.2015.10.011 26514403

[B18] Frame N. W. Allas M. J. Pequegnat B. Vinogradov E. Liao V. C.-H. Al-Abdul-Wahid S.. (2023). Structure and synthesis of a vaccine and diagnostic target for *Enterocloster bolteae*, an autism-associated gut pathogen - Part II. Carbohydr Res. 526, 108805. doi: 10.1016/j.carres.2023.108805 37023666

[B19] Gan L. Feng Y. Du B. Fu H. Tian Z. Xue G.. (2023). Bacteriophage targeting microbiota alleviates non-alcoholic fatty liver disease induced by high alcohol-producing *Klebsiella pneumoniae* . Nat. Commun. 14 (1), 3215. doi: 10.1038/s41467-023-39028-w 37270557 PMC10239455

[B20] Guo L. Wang Y. Y. Wang J. H. Zhao H.-P. Yu Y. Wang G.-D.. (2022). Associations of gut microbiota with dyslipidemia based on sex differences in subjects from Northwestern China. World J. Gastroenterol. 28 (27), 3455–3475. doi: 10.3748/wjg.v28.i27.3455 36158270 PMC9346449

[B21] Haas K. N. Blanchard J. L. (2020). Reclassification of the *Clostridium clostridioforme* and *Clostridium sphenoides* clades as *Enterocloster* gen. nov. and *Lacrimispora* gen. nov., including reclassification of 15 taxa. Int. J. Syst. Evol. Microbiol. 70 (1), 23–34. doi: 10.1099/ijsem.0.003698 31782700

[B22] Haldar D. Kern B. Hodson J. Armstrong M. J. Adam R. Berlakovich G.. (2019). Outcomes of liver transplantation for non-alcoholic steatohepatitis: A European Liver Transplant Registry study. J. Hepatol. 71 (2), 313–322. doi: 10.1016/j.jhep.2019.04.011 31071367 PMC6656693

[B23] Hall A. B. Yassour M. Sauk J.. (2017). A novel *Ruminococcus gnavus* clade enriched in inflammatory bowel disease patients. Genome Med. 9 (1), 103. doi: 10.1186/s13073-017-0490-5 29183332 PMC5704459

[B24] Hugon P. Ramasamy D. Lagier J. C.. (2013). Non contiguous-finished genome sequence and description of *Alistipes obesi* sp. nov. Stand Genomic Sci. 7 (3), 427–439. doi: 10.4056/sigs.3336746 24019990 PMC3764931

[B25] Hullar M. A. J. Jenkins I. C. Randolph T. W.. (2021). Associations of the gut microbiome with hepatic adiposity in the Multiethnic Cohort Adiposity Phenotype Study. Gut Microbes 13 (1), 1965463. doi: 10.1080/19490976.2021.1965463 34491886 PMC8425768

[B26] Hynönen U. Rasinkangas P. Satokari R. Paulin L. de Vos W. M. Pietilä T. E.. (2016). Isolation and whole genome sequencing of a *Ruminococcus*-like bacterium, associated with irritable bowel syndrome. Anaerobe 39, 60–67. doi: 10.1016/j.anaerobe.2016.03.001 26946362

[B27] Jin L. T. Xu M. Z. (2023). Characterization of gut dominant microbiota in obese patients with nonalcoholic fatty liver disease. Front. Cell Infect. Microbiol. 13. doi: 10.3389/fcimb.2023.1113643 PMC9899993 36756620

[B28] Lagier J-C. Armougom F. Million M. Hugon P. Pagnier I. Robert C.. (2012a). Microbial culturomics: paradigm shift in the human gut microbiome study. Clin. Microbiol. Infect. 18 (12), 1185–1193. doi: 10.1111/1469-0691.12023 23033984

[B29] Lagier J-C. Dubourg G. Million M. Cadoret F. Bilen M. Fenollar F.. (2018). Culturing the human microbiota and culturomics. Nat. Rev. Microbiol. 16, 540–550. doi: 10.1038/s41579-018-0041-0 29937540

[B30] Lagier J-C. Khelaifia S. Alou M. T. Ndongo S. Dione N. Hugon P.. (2016). Culture of previously uncultured members of the human gut microbiota by culturomics. Nat. Microbiol. 1, 16203. doi: 10.1038/nmicrobiol.2016.203 27819657 PMC12094094

[B31] Lagier J-C. Million M. Hugon P. Armougom F. Raoult D. (2012b). Human gut microbiota: repertoire and variations. Front. Cell Infect. Microbiol. 2. doi: 10.3389/fcimb.2012.00136 PMC3487222 23130351

[B32] Lee N. Y. Yoon S. J. Han D. H. Gupta H. Youn G. S. Shin M. J.. (2020). *Lactobacillus* and *Pediococcus* ameliorate progression of non-alcoholic fatty liver disease through modulation of the gut microbiome. Gut Microbes 11 (4), 882–899. doi: 10.1080/19490976.2020.1712984 31965894 PMC7524267

[B33] Lin D. Sun Q. Liu Z.. (2023). Gut microbiota and bile acids partially mediate the improvement of fibroblast growth factor 21 on methionine-choline-deficient diet-induced non-alcoholic fatty liver disease mice. Free Radic. Biol. Med. 195, 199–218. doi: 10.1016/j.freeradbiomed.2022.12.087 36586452

[B34] Loomba R. Seguritan V. Li W. Long T. Klitgord N. Bhatt A.. (2019). Gut microbiome-based metagenomic signature for non-invasive detection of advanced fibrosis in human nonalcoholic fatty liver disease. Cell Metab. 30 (3), 607. doi: 10.1016/j.cmet.2019.08.002 31484056 PMC8025688

[B35] Magdy Wasfy R. Mbaye B. Borentain P. Tidjani Alou M. Murillo Ruiz M. L. Caputo A.. (2023). Ethanol-producing *enterocloster bolteae* is enriched in chronic hepatitis B-associated gut dysbiosis: A case–control culturomics study. Microorganisms 11 (10), 2437. doi: 10.3390/microorganisms11102437 37894093 PMC10608849

[B36] Martens K. De Boeck I. Jokicevic K. Kiekens F. Farré R. Vanderveken O. M.. (2022). Erratum: *lacticaseibacillus casei* AMBR2 restores airway epithelial integrity in chronic rhinosinusitis with nasal polyps. Allergy Asthma Immunol. Res. 14 (1), 146. doi: 10.4168/aair.2022.14.1.146 34983115 PMC8724824

[B37] Mbaye B. Borentain P. Magdy Wasfy R. Alou M. T. Armstrong N. Mottola G.. (2022). Endogenous Ethanol and Triglyceride Production by Gut *Pichia kudriavzevii*, *Candida albicans* and *Candida glabrata* Yeasts in Non-Alcoholic Steatohepatitis. Cells 11 (21), 3390. doi: 10.3390/cells11213390 36359786 PMC9654979

[B38] Mbaye B. Wasfy R. M. Alou M. T. Borentain P. Andrieu C. Caputo A.. (2023). Limosilactobacillus fermentum, Lactococcus lactis and Thomasclavelia ramosa are enriched and Methanobrevibacter smithii is depleted in patients with non-alcoholic steatohepatitis. Microb Pathog 180, 106160. doi: 10.1016/j.micpath.2023.106160 37217120

[B39] Meijnikman A. S. Davids M. Herrema H. Aydin O. Tremaroli V. Rios-Morales M.. (2022). Microbiome-derived ethanol in nonalcoholic fatty liver disease. Nat. Med. 28 (10), 2100–2106. doi: 10.1038/s41591-022-02016-6 36216942

[B40] Million M. Maraninchi M. Henry M. Armougom F. Richet H. Carrieri P.. (2012). Obesity-associated gut microbiota is enriched in *Lactobacillus reuteri* and depleted in *Bifidobacterium animalis* and Methanobrevibacter smithii. Int. J. Obes. (Lond) 36 (6), 817–825. doi: 10.1038/ijo.2011.153 21829158 PMC3374072

[B41] Mohan R. Namsolleck P. Lawson P. A. Osterhoff M. Collins M. D. Alpert C.-A.. (2006). *Clostridium asparagiforme* sp. nov., isolated from a human faecal sample. Syst. Appl. Microbiol. 29 (4), 292–299. doi: 10.1016/j.syapm.2005.11.001 16337765

[B42] Naka S. Nomura R. Takashima Y. Okawa R. Ooshima T. Nakano K. (2014). A specific *Streptococcus mutans* strain aggravates non-alcoholic fatty liver disease. Oral. Dis. 20 (7), 700–706. doi: 10.1111/odi.12191 25360469

[B43] Paik J. M. Golabi P. Younossi Y. Mishra A. Younossi Z. M. (2020). Changes in the global bur-den of chronic liver diseases from 2012 to 2017: the growing impact of NAFLD. Hepatology 72 (5), 1605–1616. doi: 10.1002/hep.31173 32043613

[B44] Pandit L. Cox L. M. Malli C. D’Cunha A. Rooney T. Lokhande H.. (2021). *Clostridium bolteae* is elevated in neuromyelitis optica spectrum disorder in India and shares sequence similarity with AQP4. Neurol. Neuroimmunol Neuroinflamm 8, e907. doi: 10.1212/NXI.0000000000000907 33148687 PMC7643530

[B45] Pham T.-P.-T. Tidjani Alou M. Bachar D. Levasseur A. Brah S. Alhousseini D.. (2019). Gut microbiota alteration is characterized by a *proteobacteria* and *fusobacteria* bloom in kwashiorkor and a bacteroidetes paucity in marasmus. Sci. Rep. 9 (1), 9084. doi: 10.1038/s41598-019-45611-3 31235833 PMC6591176

[B46] Ruuskanen M. O. Åberg F. Männistö V. Havulinna A. S. Méric G. Liu Y.. (2021). Links between gut microbiome composition and fatty liver disease in a large population sample. Gut Microbes 13 (1), 1–22. doi: 10.1080/19490976.2021.188867 PMC7928040 33651661

[B47] Ruuskanen M. O. Erawijantari P. P. Havulinna A. S. Liu Y. Méric G. Tuomilehto J.. (2022). Gut microbiome composition is predictive of incident type 2 diabetes in a population cohort of 5,572 finnish adults. Diabetes Care 45 (4), 811–818. doi: 10.2337/dc21-2358 35100347 PMC9016732

[B48] Samuel B. S. Gordon J. I. (2006). A humanized gnotobiotic mouse model of host-archaeal-bacterial mutualism. Proc. Natl. Acad. Sci. U S A. 103 (26), 10011–10016. doi: 10.1073/pnas.0602187103 16782812 PMC1479766

[B49] Song Y. Liu C. Molitoris D. R. Tomzynski T. J. Lawson P. A. Collins M. D.. (2003). *Clostridium bolteae* sp. nov., isolated from human sources. Syst. Appl. Microbiol. 26 (1), 84–89. doi: 10.1078/072320203322337353 12747414

[B50] Song Q. Zhang X. Liu W. Wei H. Liang W. Zhou Y.. (2023). Bifidobacterium pseudolongum-generated acetate suppresses non-alcoholic fatty liver disease-associated hepatocellular carcinoma. J. Hepatol. S0168-8278 (23), 04981–04984. doi: 10.1016/j.jhep.2023.07.005 37459922

[B51] Spanhaak S. Havenaar R. Schaafsma G. (1998). The effect of consumption of milk fermented by *Lactobacillus casei* strain Shirota on the intestinal microflora and immune parameters in humans. Eur. J. Clin. Nutr. 52 (12), 899–907. doi: 10.1038/sj.ejcn.1600663 9881885

[B52] Takahashi N. Iwami Y. Yamada T. (1991). Metabolism of intracellular polysaccharide in the cells of *Streptococcus mutans* under strictly anaerobic conditions. Oral. Microbiol. Immunol. 6 (5), 299–304. doi: 10.1111/j.1399-302x.1991.tb00497.x 1820569

[B53] Tidjani Alou M. Million M. Traore S. I. Mouelhi D. Khelaifia S. Bachar D.. (2017). Gut bacteria missing in severe acute malnutrition, can we identify potential probiotics by culturomics? Front. Microbiol. 8. doi: 10.3389/fmicb.2017.00899 PMC5440526 28588566

[B54] Tidjani Alou M. Naud S. Khelaifia S. Bonnet M. Lagier J. C. Raoult D. (2020). State of the art in the culture of the human microbiota: new interests and strategies. Clin. Microbiol. Rev. 34 (1), e00129–e00119. doi: 10.1128/CMR.00129-19 33115723 PMC7605308

[B55] Togo A. H. Diop A. Bittar F. Maraninchi M. Valero R. Armstrong N.. (2018). Description of *Mediterraneibacter massiliensis*, gen. nov., sp. nov., a new genus isolated from the gut microbiota of an obese patient and reclassification of *Ruminococcus faecis*, *Ruminococcus lactaris*, *Ruminococcus torques*, *Ruminococcus gnavus* and *Clostridium glycyrrhizinilyticum* as *Mediterraneibacter* faecis comb. nov., *Mediterraneibacter lactaris* comb. nov., Mediterraneibacter torques comb. nov., *Mediterraneibacter gnavus* comb. nov. and *Mediterraneibacter glycyrrhizinilyticus* comb. nov. Antonie Van Leeuwenhoek. 111 (11), 2129–2130. doi: 10.1007/s10482-018-1171-0 30267233

[B56] Vernekar M. Singhal R. Joshi K. Amarapurkar D. (2018). Variation in the plasma levels of polyunsaturated fatty acids in control vis-à-vis nonalcoholic fatty liver disease subjects and its possible association with gut microbiome. Metab. Syndr. Relat. Disord. 16 (7), 329−35. doi: 10.1089/met.2018.0008 29873593

[B57] World Medical Association (2013). World Medical Association Declaration of Helsinki: ethical principles for medical research involving human subjects. JAMA 310 (20), 2191–2194. doi: 10.1001/jama.2013.281053 24141714

[B58] Wu F. Guo X. Zhang J. Zhang M. Ou Z. Peng Y. (2017). *Phascolarctobacterium faecium* abundant colonization in human gastrointestinal tract. Exp. Ther. Med. 14 (4), 3122–3126. doi: 10.3892/etm.2017.4878 28912861 PMC5585883

[B59] Wu J. Yang K. Fan H. Wei M. Xiong Q. (2023). Targeting the gut microbiota and its metabolites for type 2 diabetes mellitus. Front. Endocrinol. (Lausanne). 14. doi: 10.3389/fendo.2023.1114424 PMC10204722 37229456

[B60] Younossi Z. Anstee Q. M. Marietti M. Hardy T. Henry L. Eslam M.. (2018). Global burden of NAFLD and NASH: trends, predictions, risk factors and prevention. Nat. Rev. Gastroenterol. Hepatol. 15 (1), 11–20. doi: 10.1038/nrgastro.2017.109 28930295

[B61] Younossi Z. M. Koenig A. B. Abdelatif D. Fazel Y. Henry L. Wymer M. (2016). Global epidemiology of nonalcoholic fatty liver disease. Meta-analytic Assess. prevalence incidence outcomes. Hepatology. 64 (1), 73–84. doi: 10.1002/hep.28431 26707365

[B62] Yuan J. Chen C. Cui J. Lu J. Yan C. Wei X.. (2019). Fatty liver disease caused by high-alcohol-producing klebsiella pneumoniae. Cell Metab. 30 (4), 675–688.e7. doi: 10.1016/j.cmet.2019.08.018 31543403

[B63] Yuan X. Zhang Y. Lin X. Yang X. Chen R. (2023). Association of gut microbiota and glucose metabolism in children with disparate degrees of adiposity. Pediatr. Obes. 18 (4), e13009. doi: 10.1111/ijpo.13009 36704910

[B64] Zheng J. Wittouck S. Salvetti E. Franz C. M. A. P. Harris H. M. B. Mattarelli P.. (2020). A taxonomic note on the genus *Lactobacillus*: Description of 23 novel genera, emended description of the genus *Lactobacillus* Beijerinck 1901, and union of Lactobacillaceae and *Leuconostocaceae* . Int. J. SystEvolMicrobiol. 70 (4), 2782−858. doi: 10.1099/ijsem.0.004107 32293557

[B65] Zhu L. Baker S. S. Gill C. Liu W. Alkhouri R. Baker R. D.. (2013). Characterization of gut microbiomes in nonalcoholic steatohepatitis (NASH) patients: a connection between endogenous alcohol and NASH. Hepatol. Baltim Md. 57 (2), 601–9. doi: 10.1002/hep.26093 23055155

