# Supplementary material for: Increased fecal ethanol and enriched ethanol-producing gut bacteria Limosilactobacillus fermentum, Enterocloster bolteae, Mediterraneibacter gnavus and Streptococcus mutans in nonalcoholic steatohepatitis
Source: Front Cell Infect Microbiol. 2023 Nov 16;13:1279354. doi: 10.3389/fcimb.2023.1279354 (PMC10687429; doi:10.3389/fcimb.2023.1279354)
Supplement: Supplementary file 1 [file Presentation_1.pptx]

## Slide 1
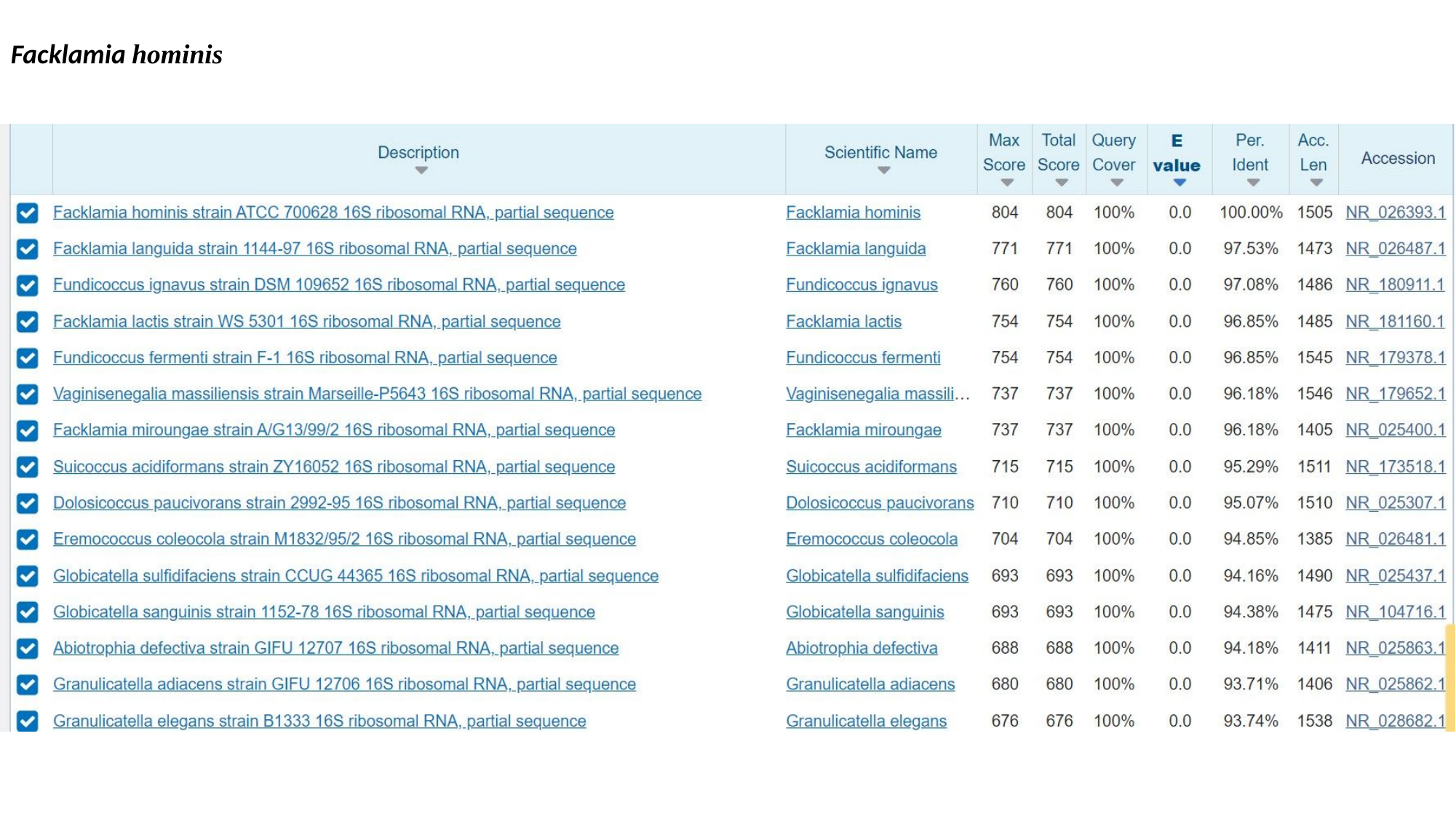

Facklamia hominis

## Slide 2
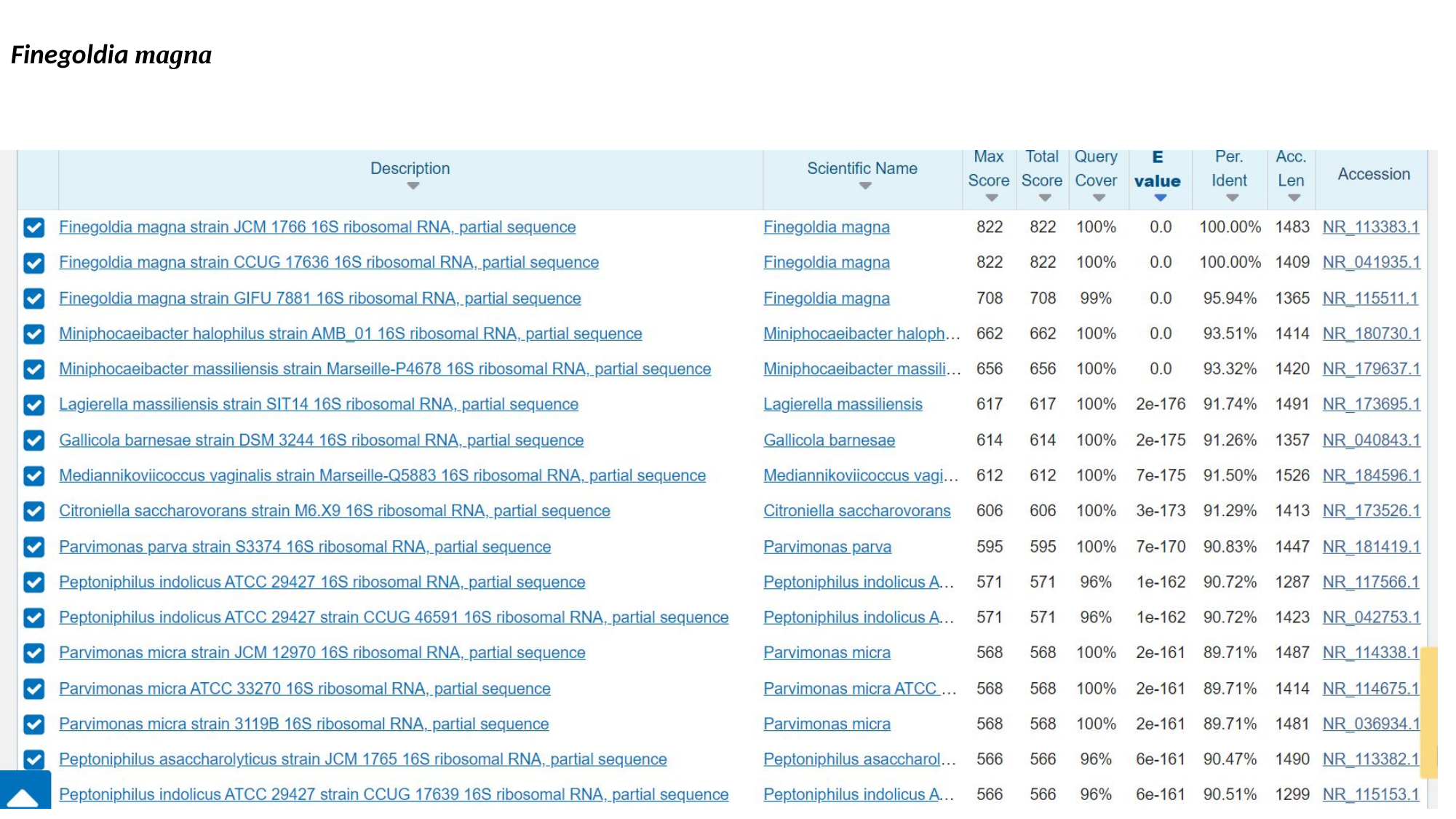

Finegoldia magna

## Slide 3
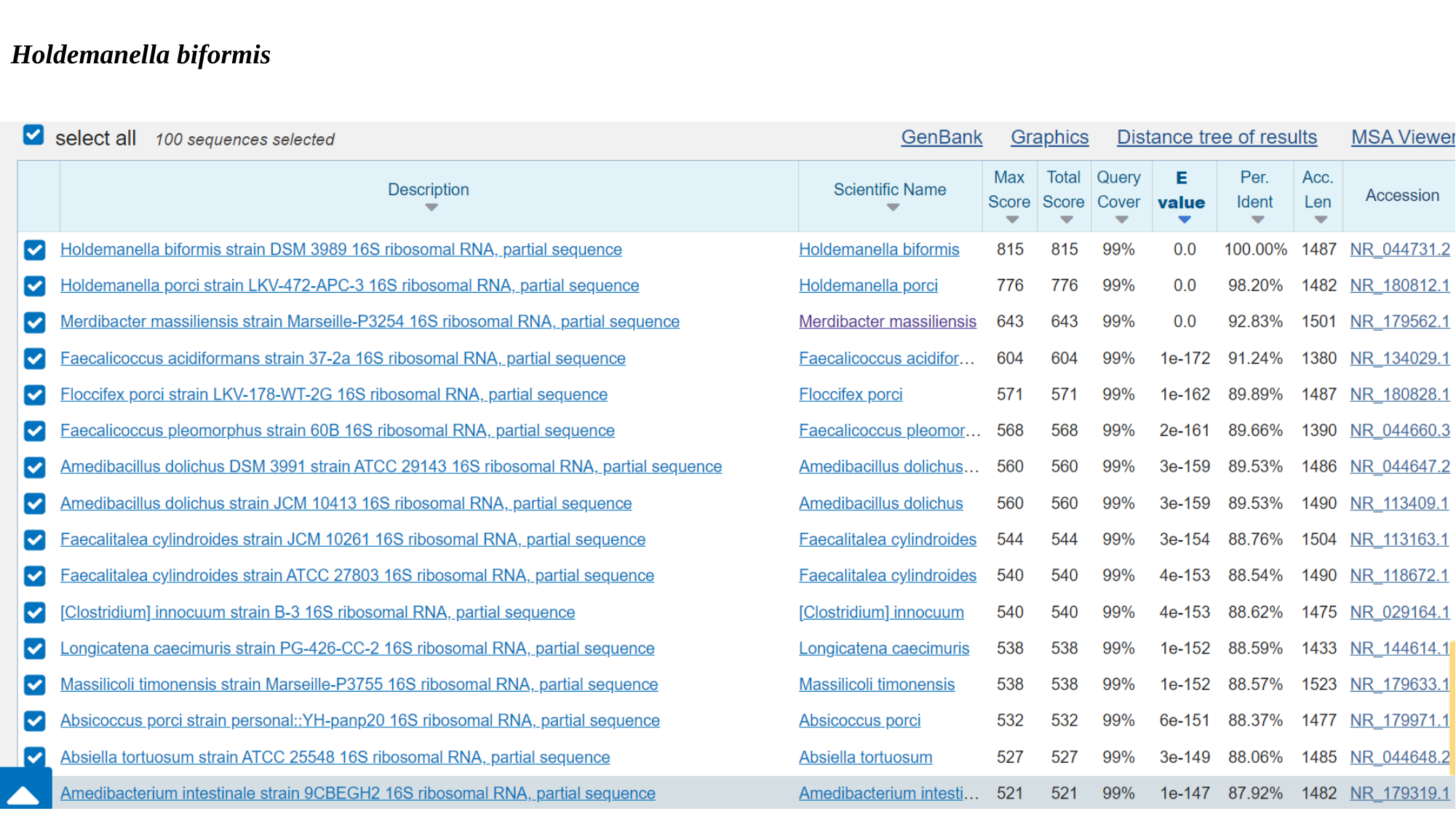

Holdemanella biformis

## Slide 4
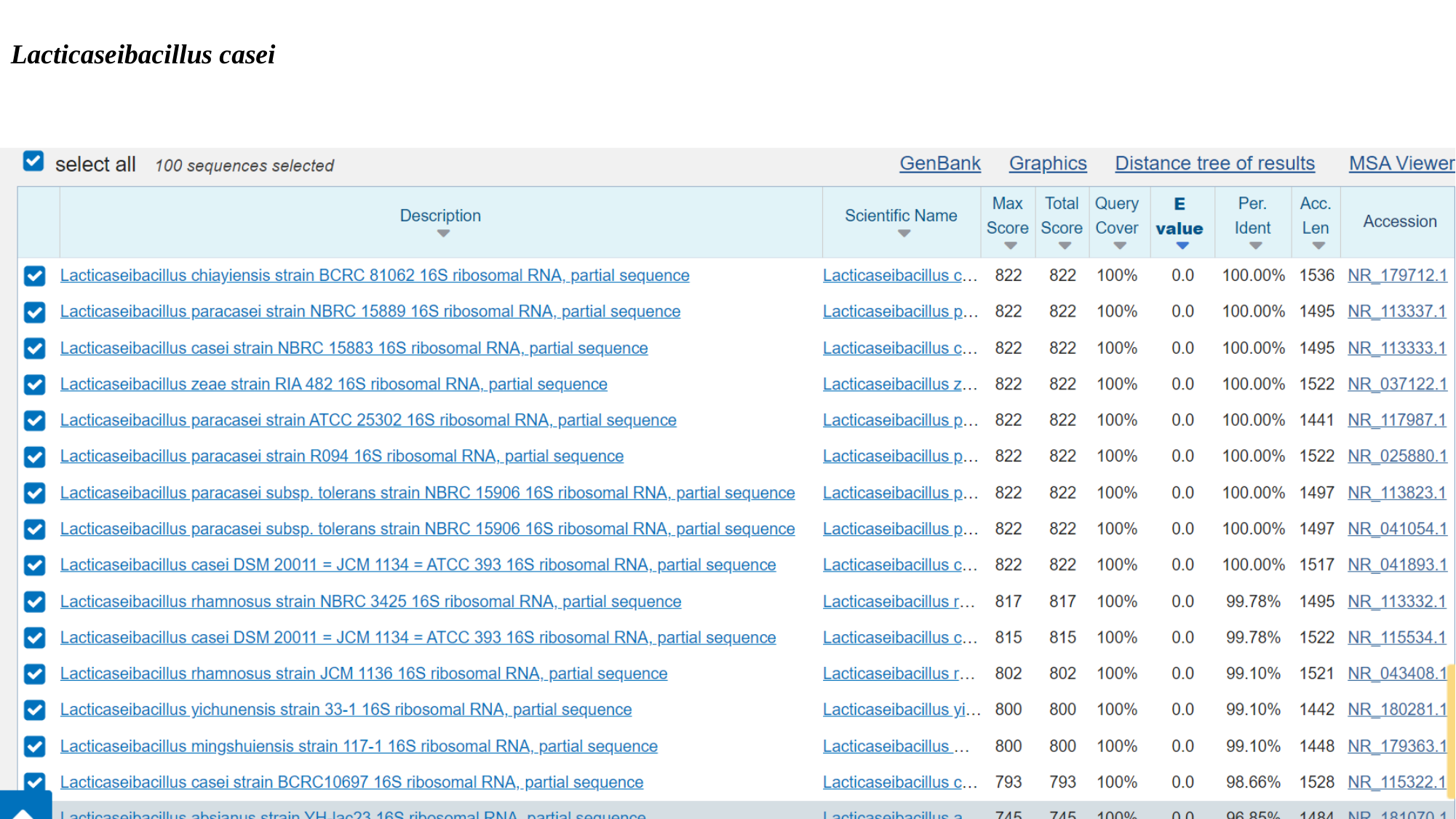

Lacticaseibacillus casei

## Slide 5
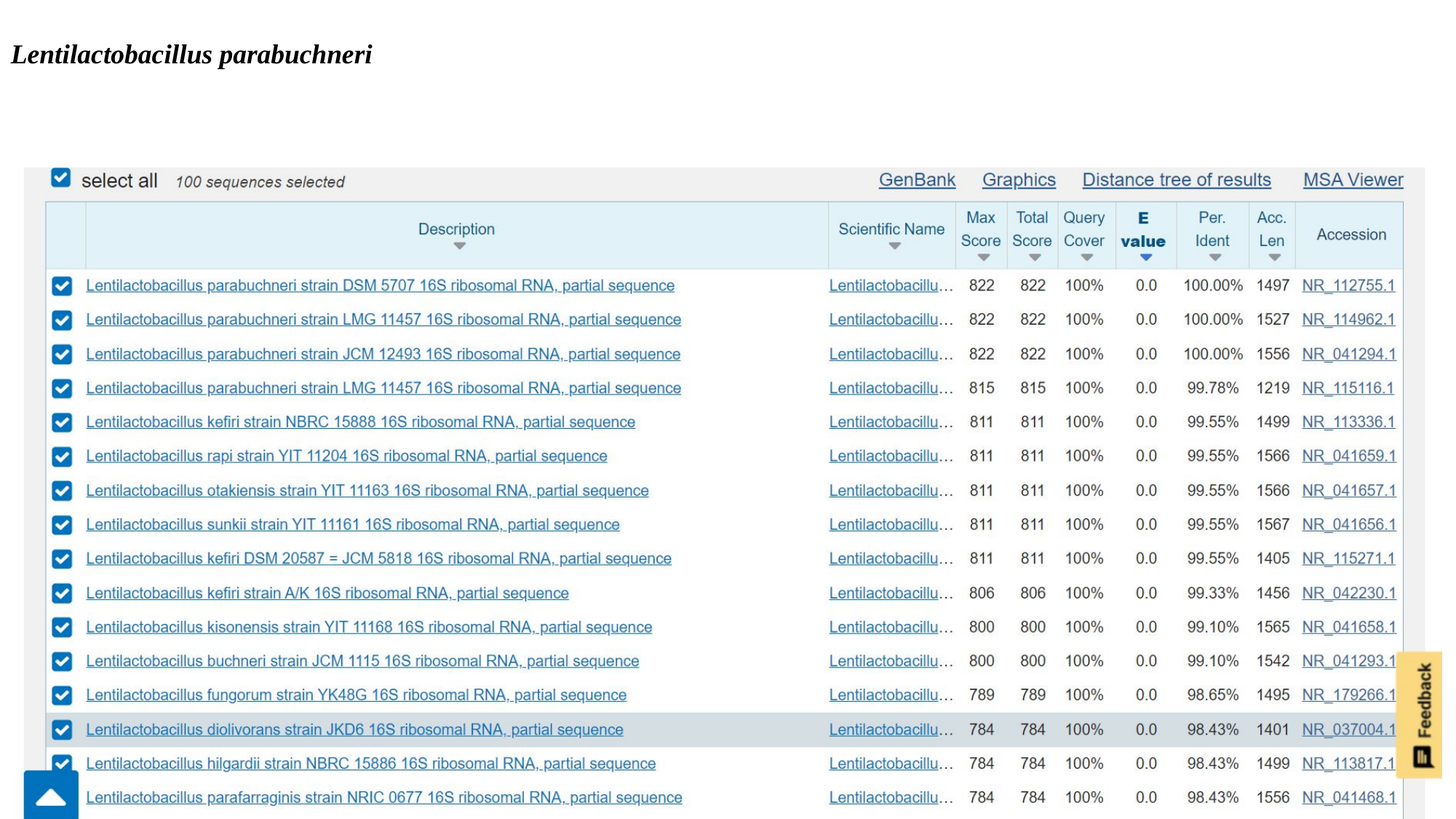

Lentilactobacillus parabuchneri

## Slide 6
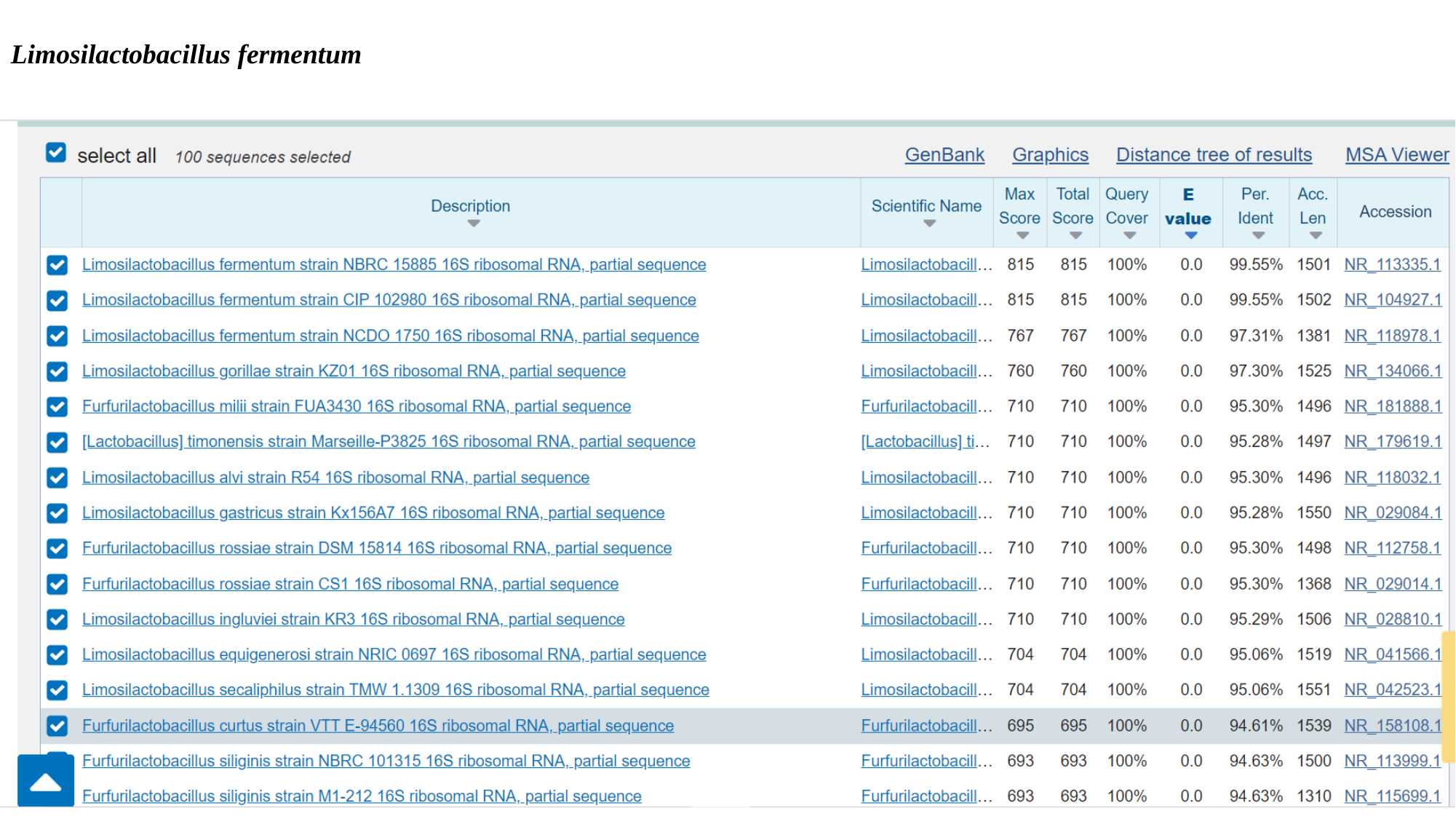

Limosilactobacillus fermentum

## Slide 7
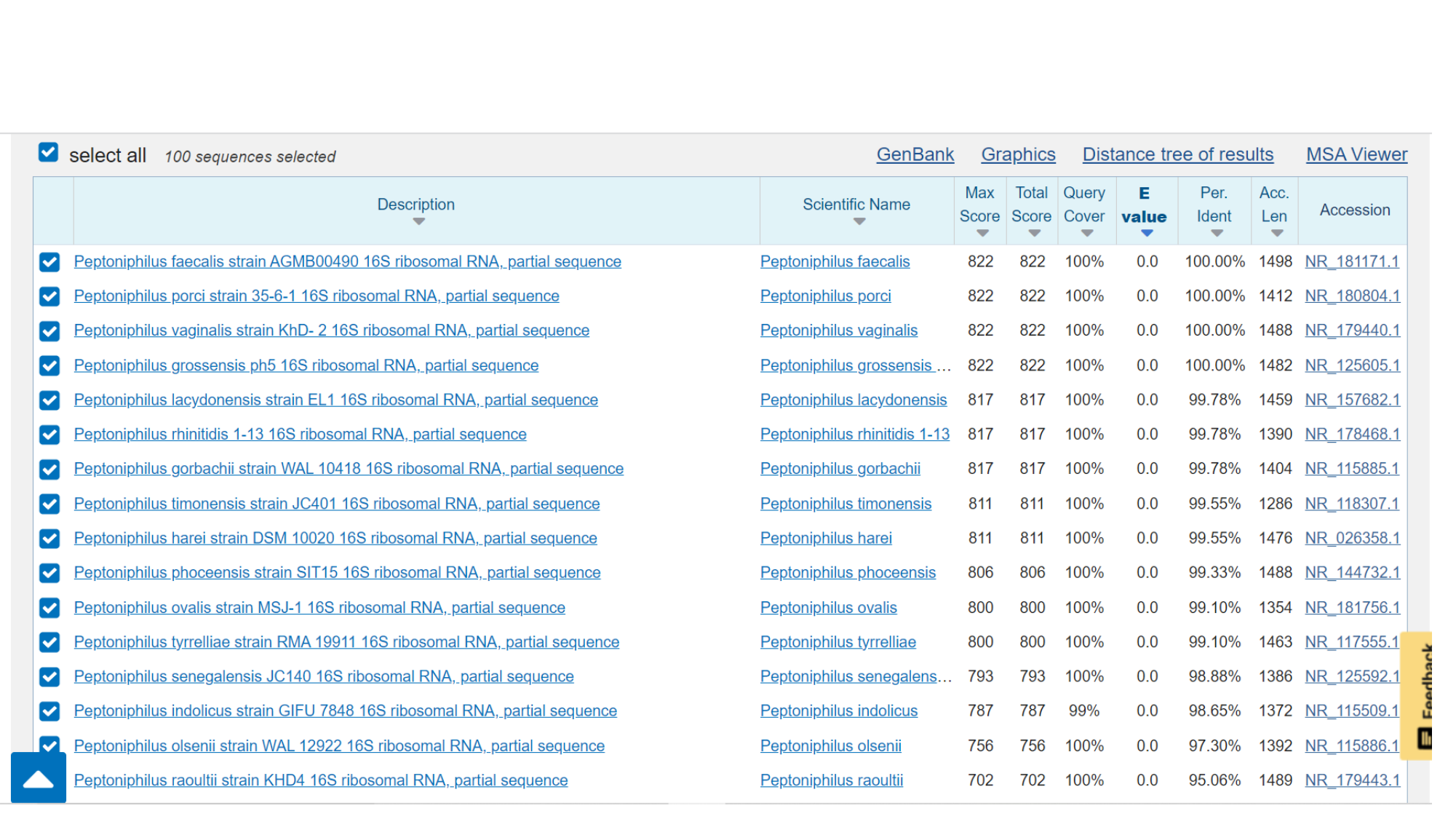

## Slide 8
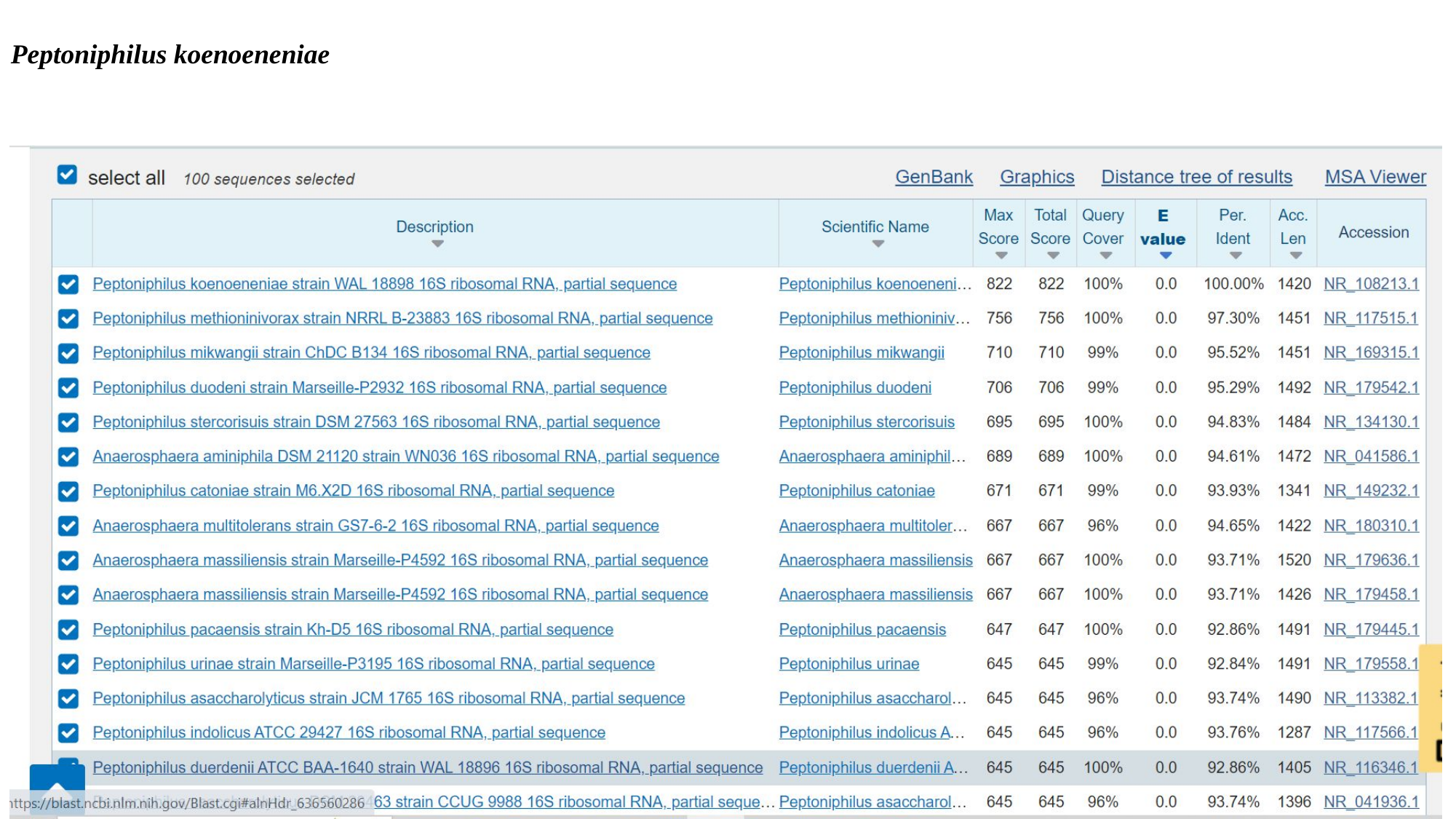

Peptoniphilus koenoeneniae

## Slide 9
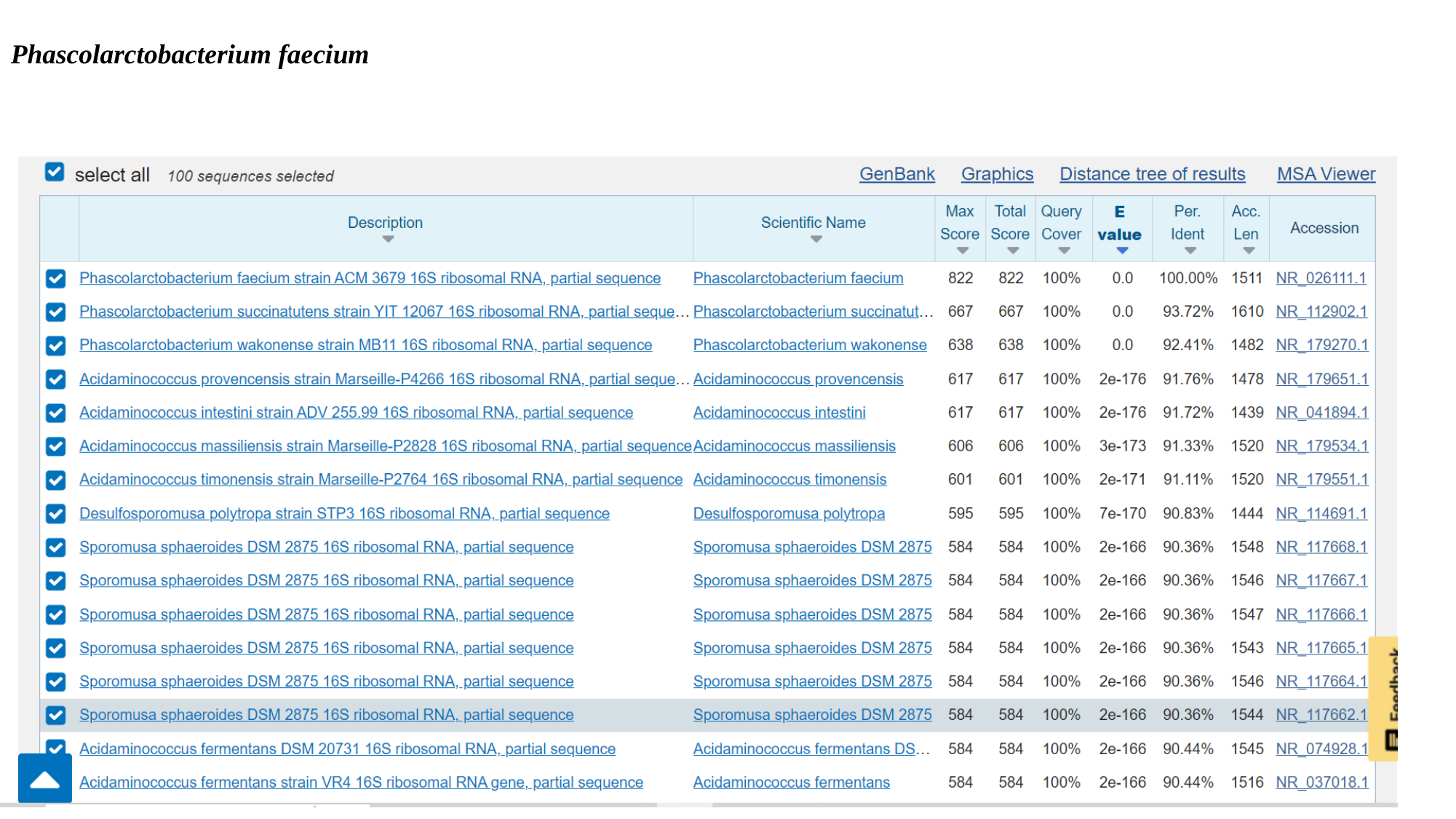

Phascolarctobacterium faecium

## Slide 10
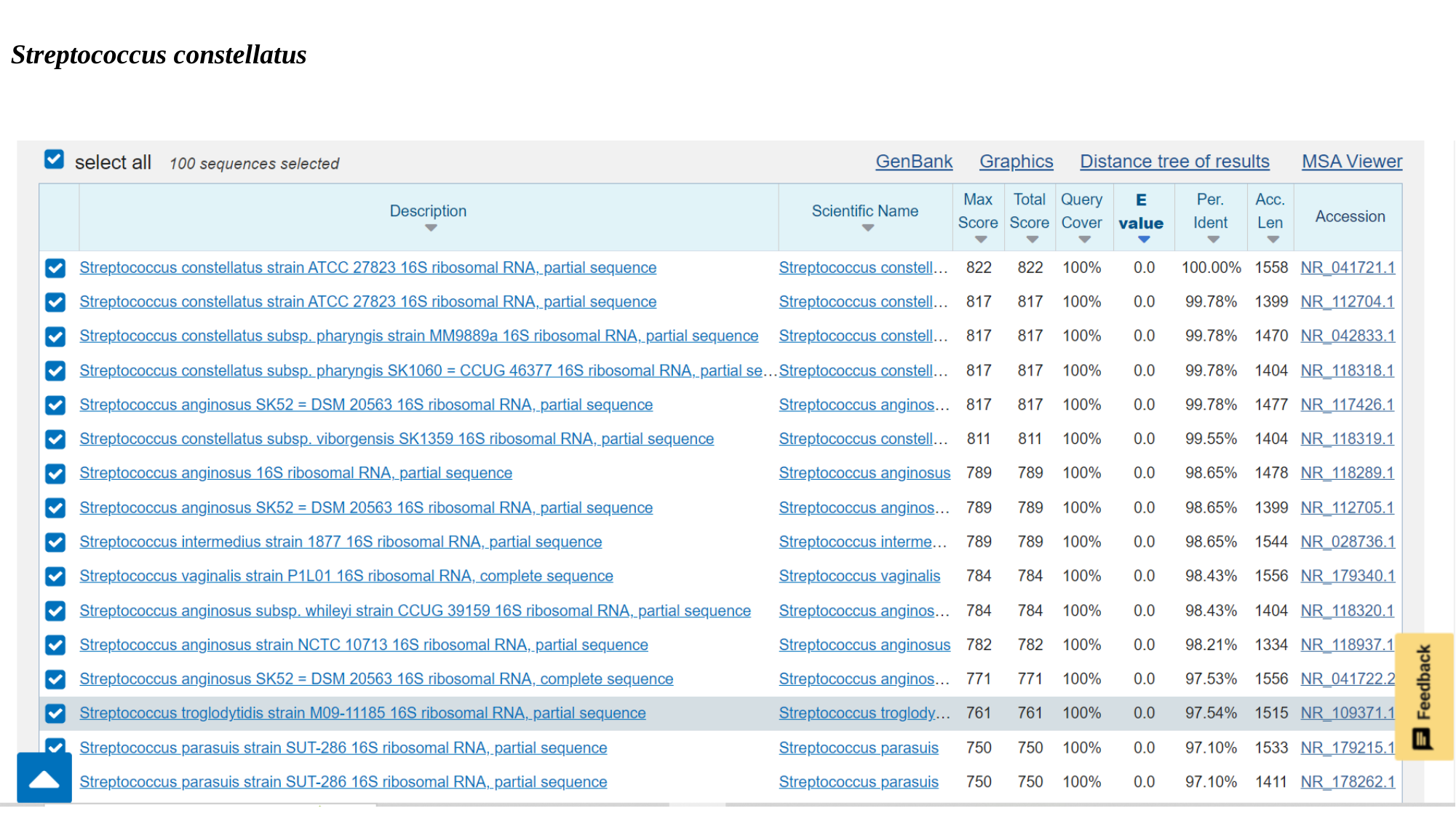

Streptococcus constellatus

## Slide 11
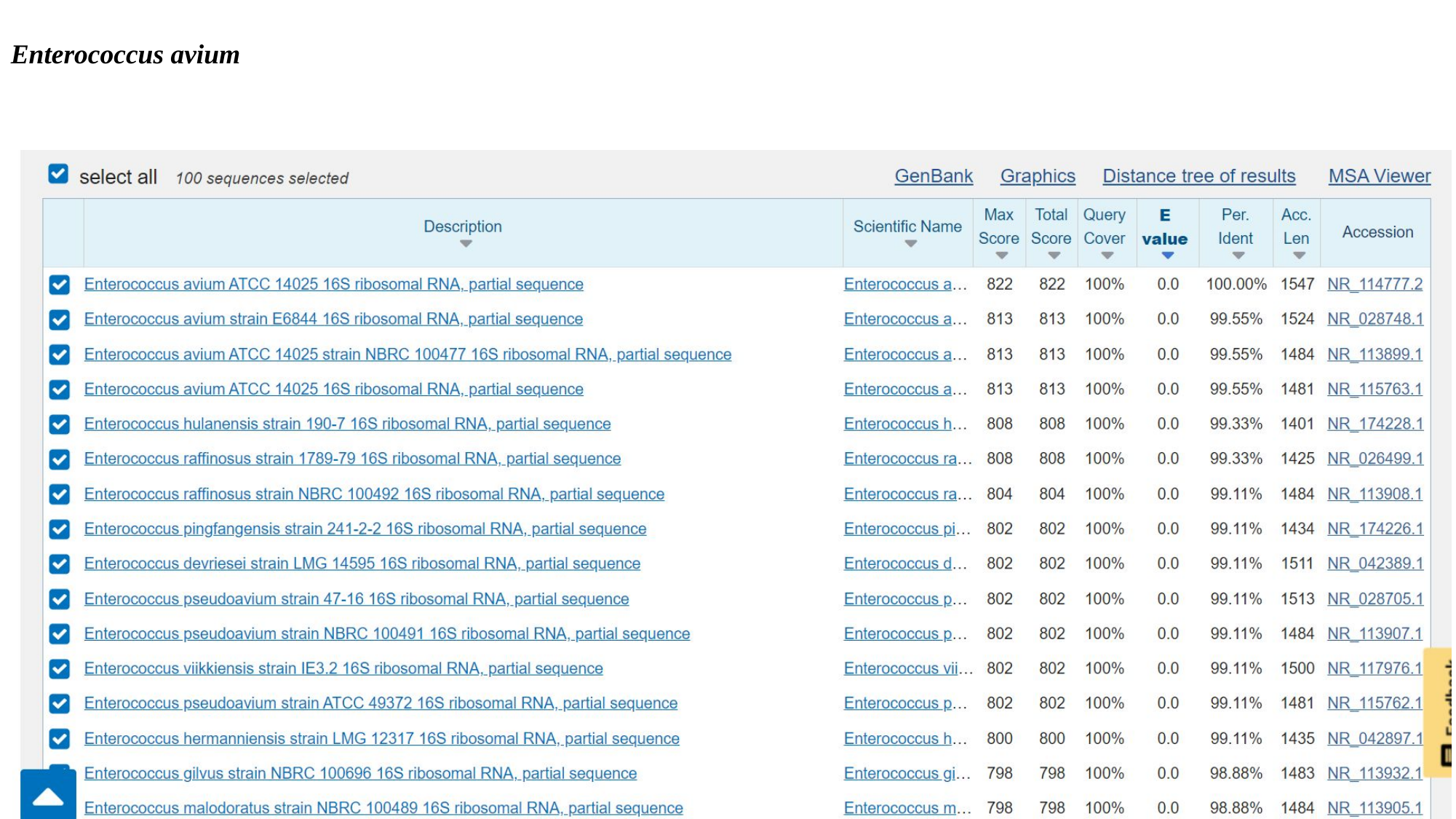

Enterococcus avium

## Slide 12
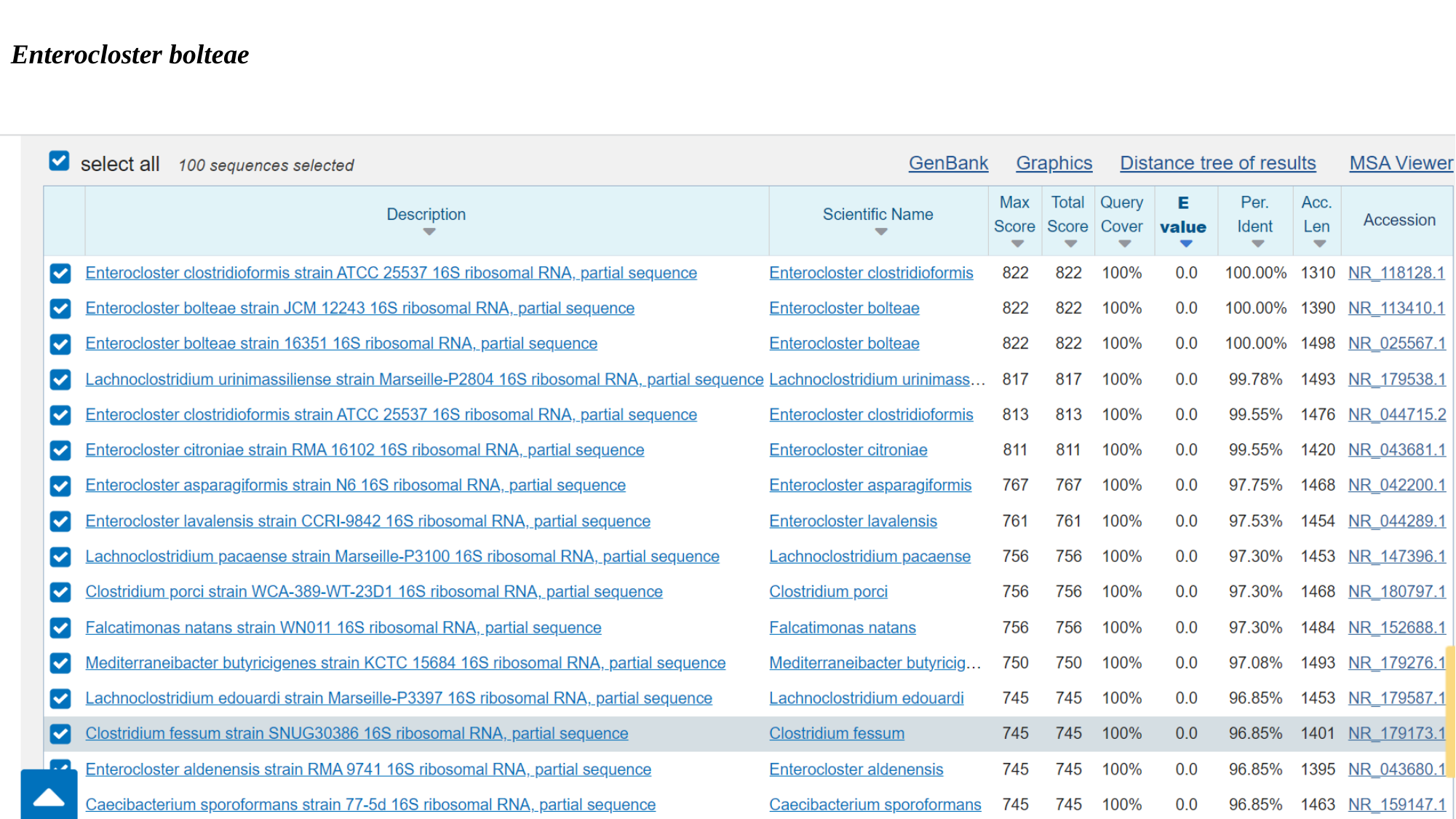

Enterocloster bolteae

## Slide 13
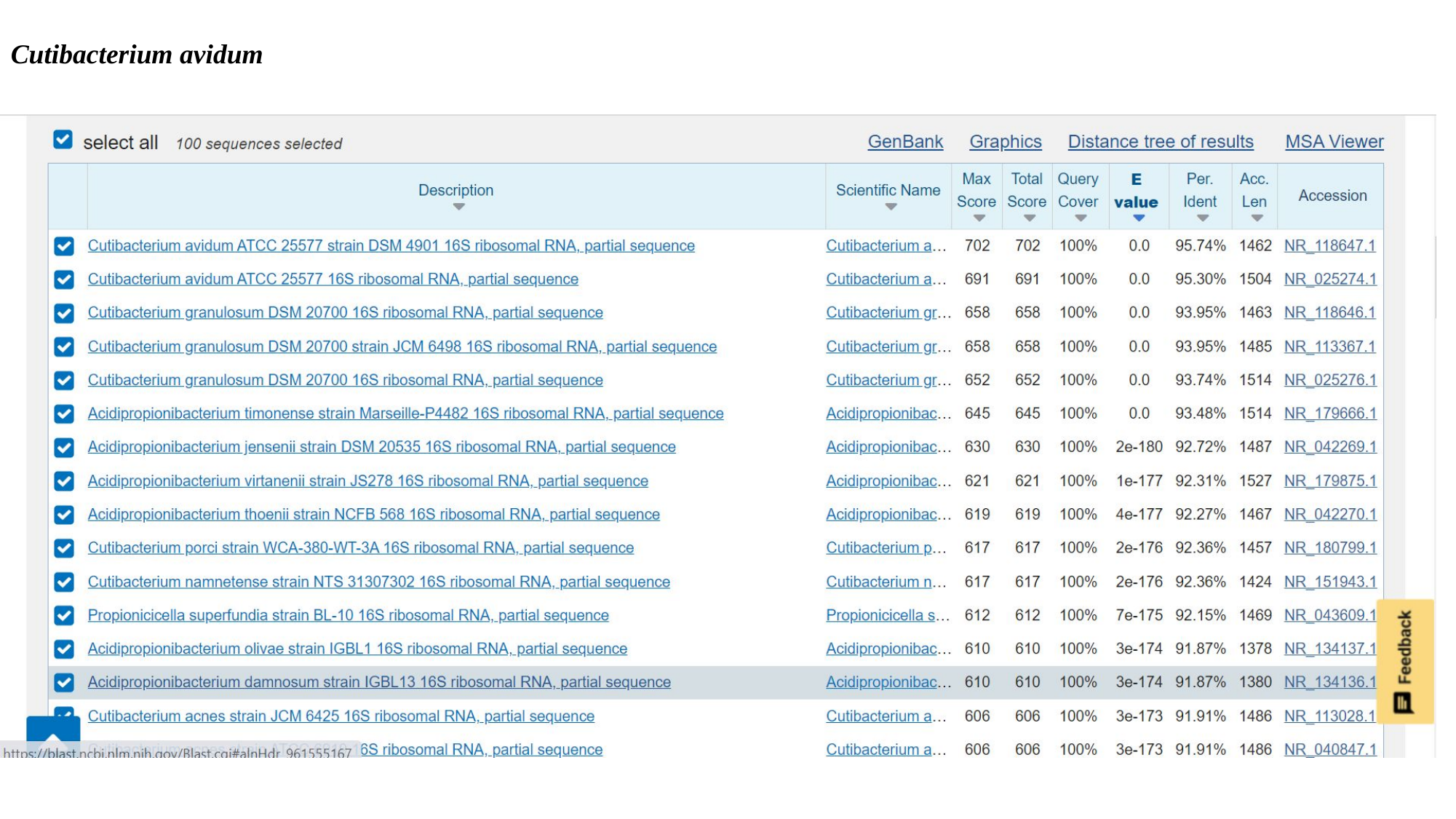

Cutibacterium avidum

## Slide 14
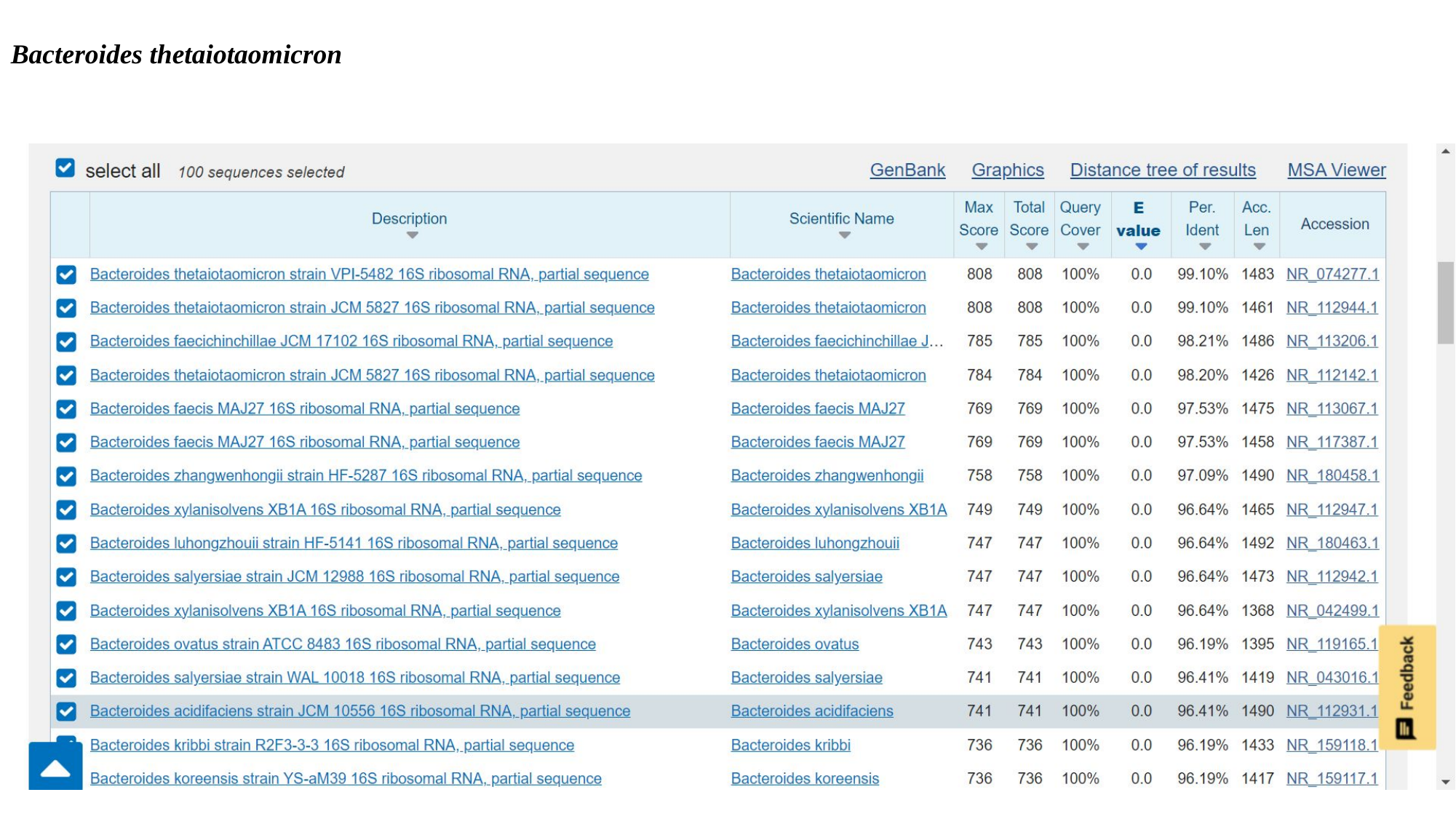

Bacteroides thetaiotaomicron

## Slide 15
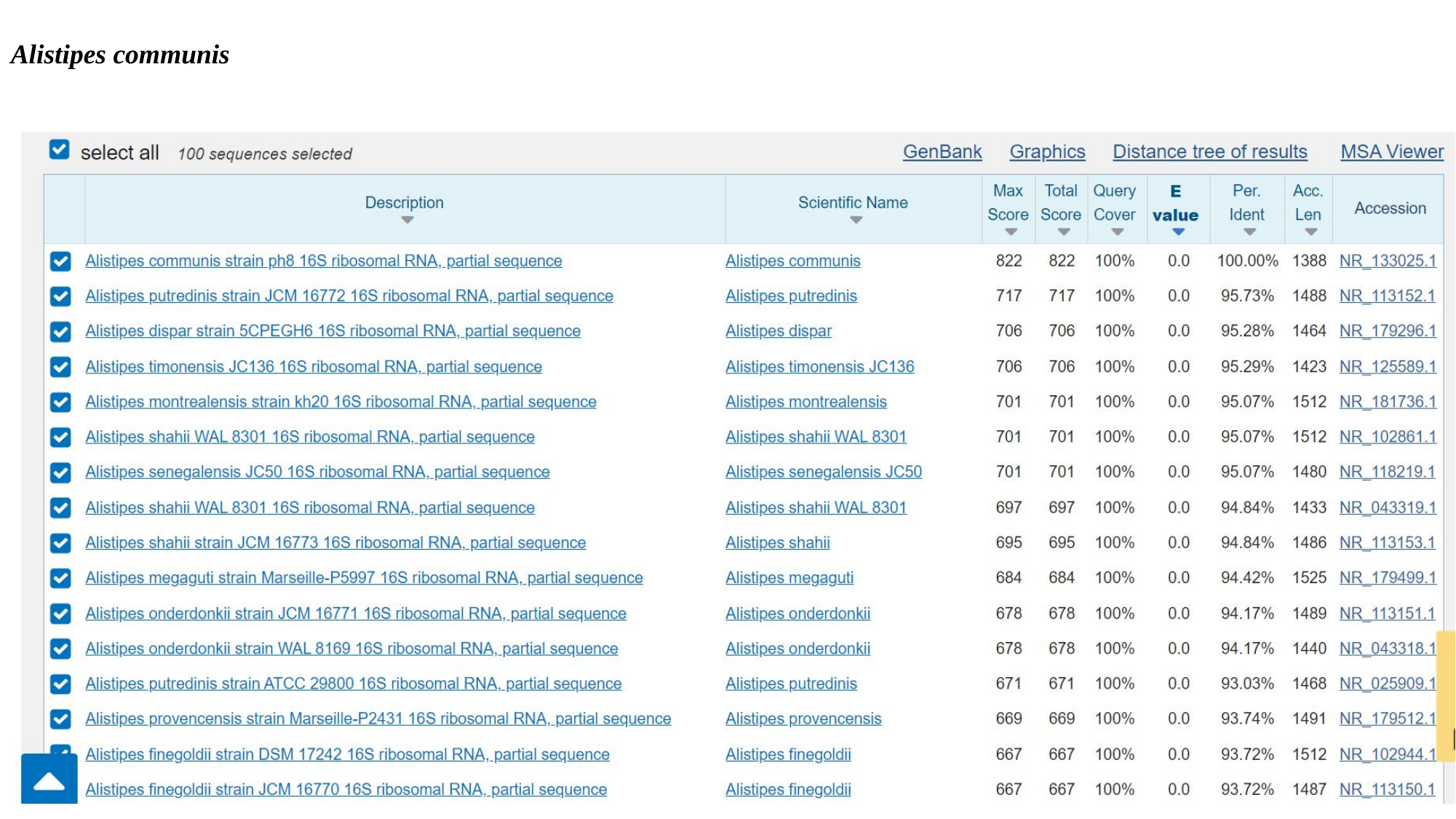

Alistipes communis

## Slide 16
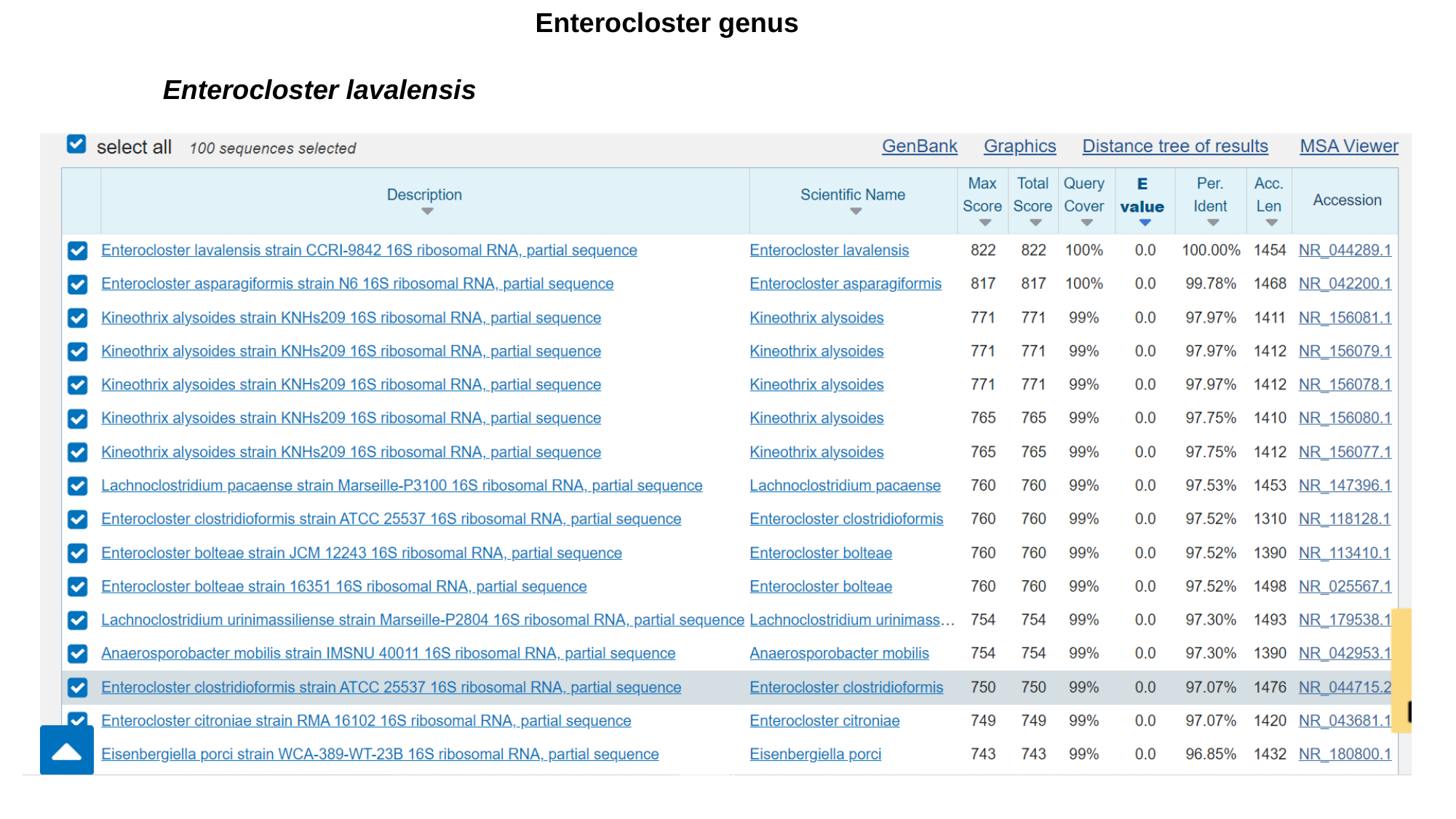

Enterocloster genus
Enterocloster lavalensis

## Slide 17
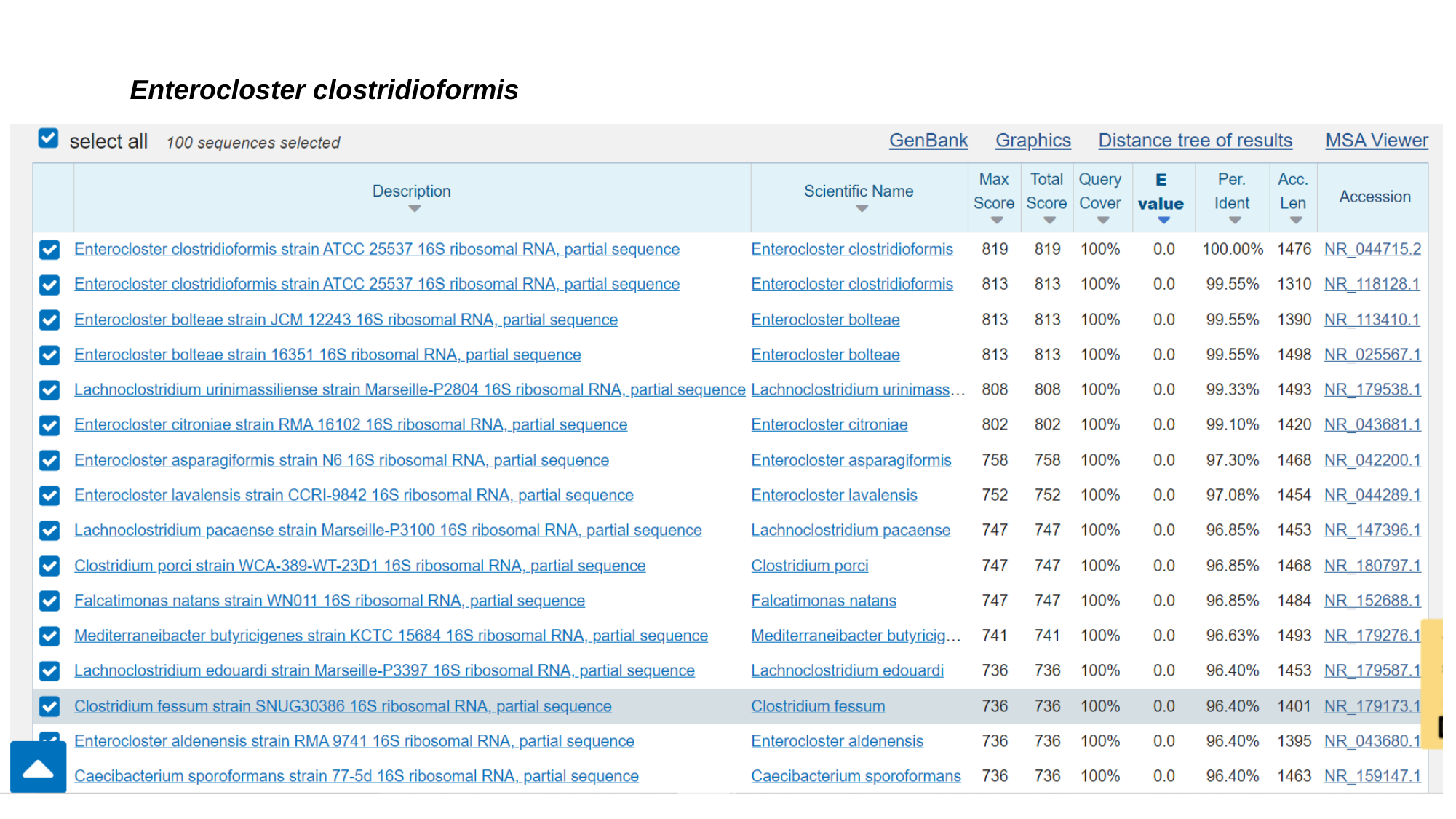

Enterocloster clostridioformis

## Slide 18
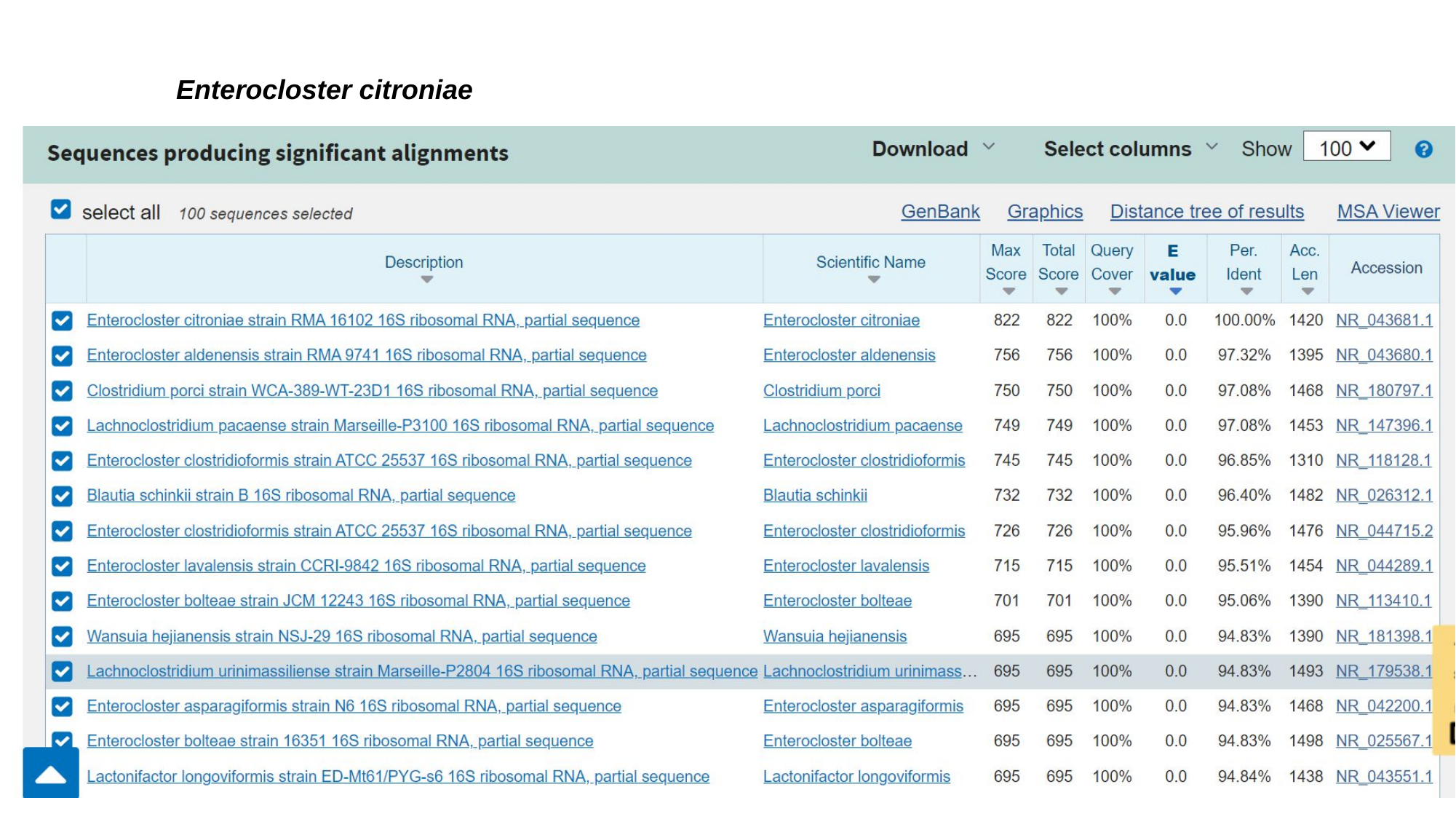

Enterocloster citroniae

## Slide 19
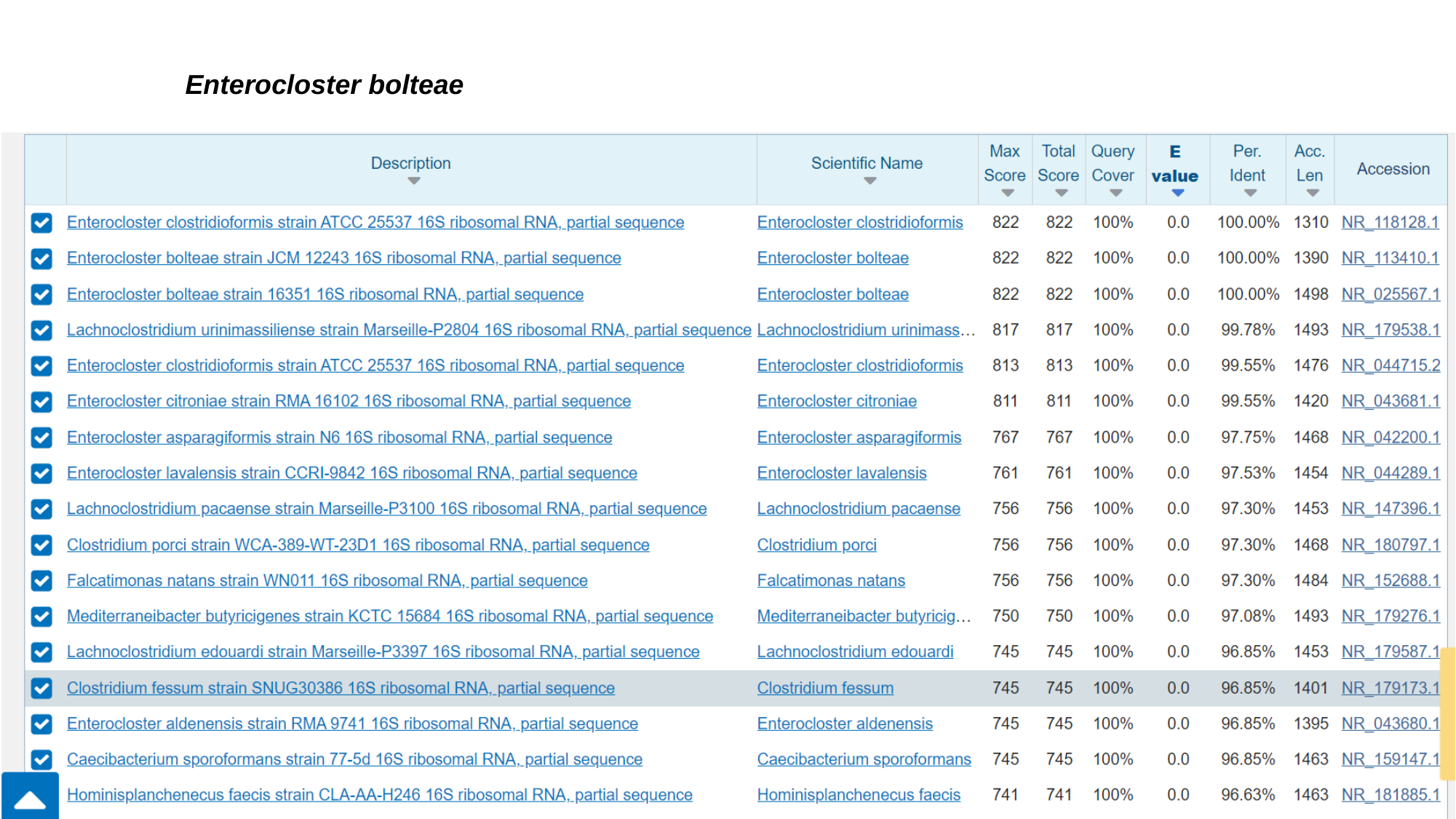

Enterocloster bolteae

## Slide 20
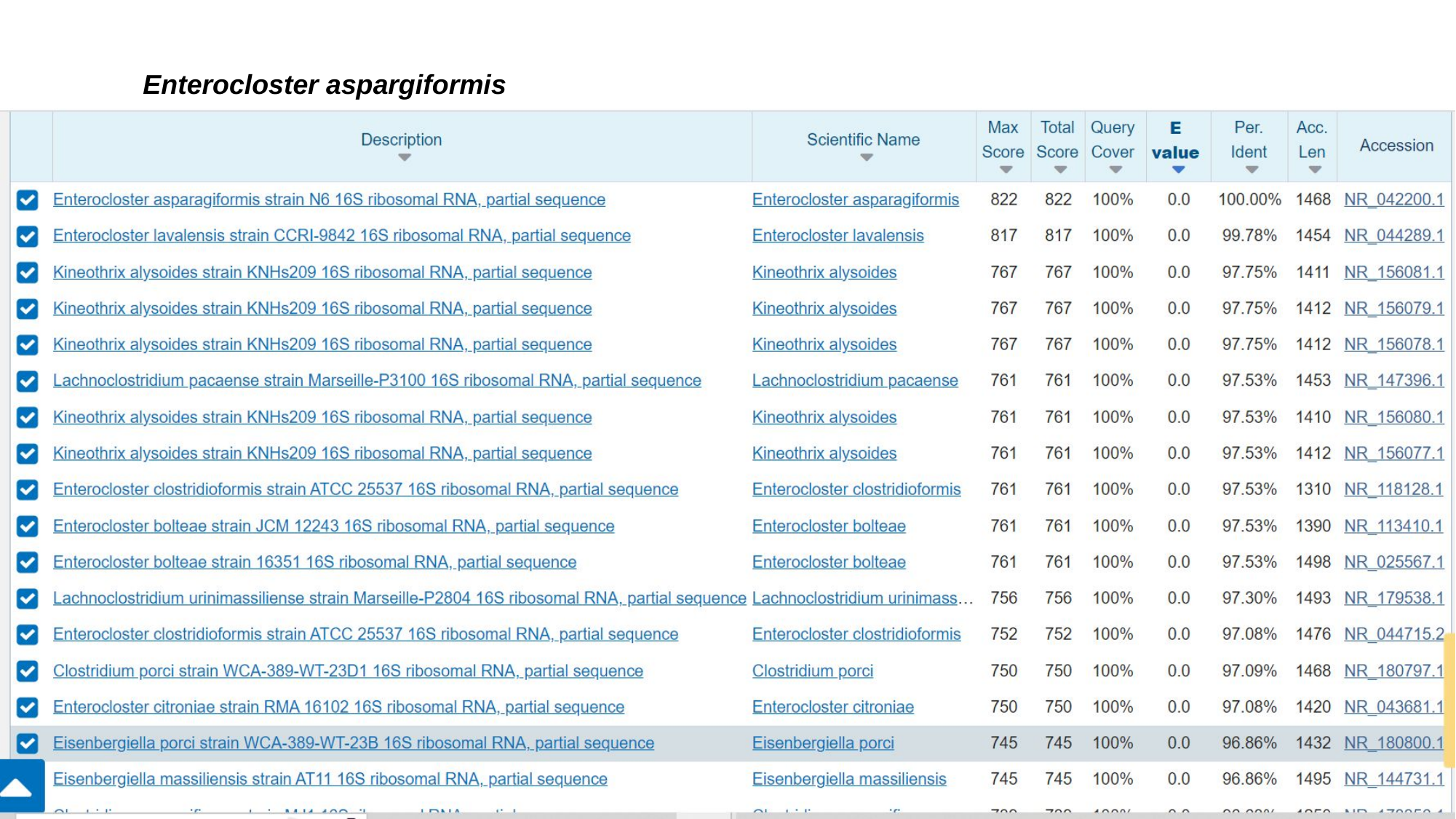

Enterocloster aspargiformis

## Slide 21
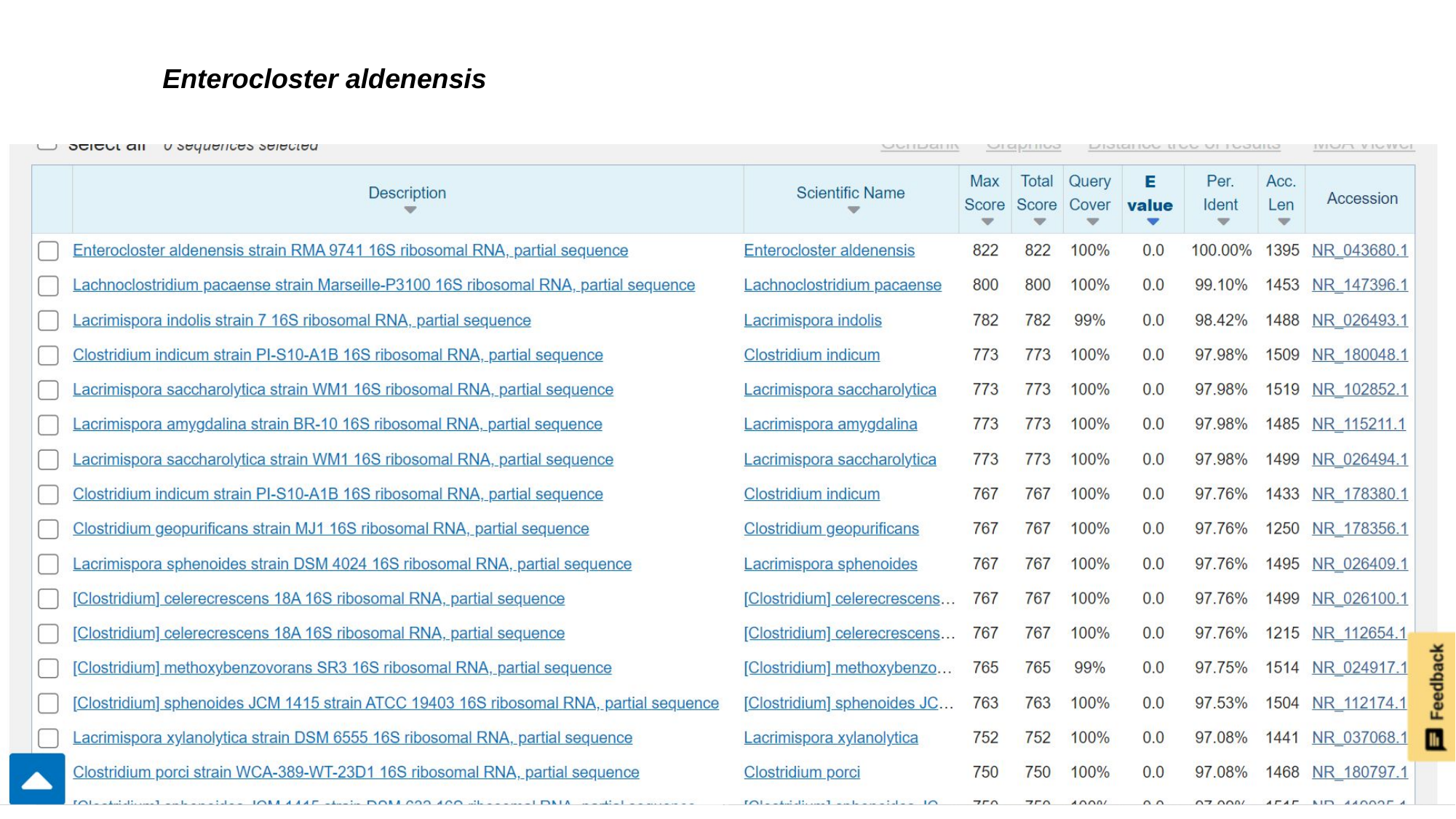

Enterocloster aldenensis

## Slide 22
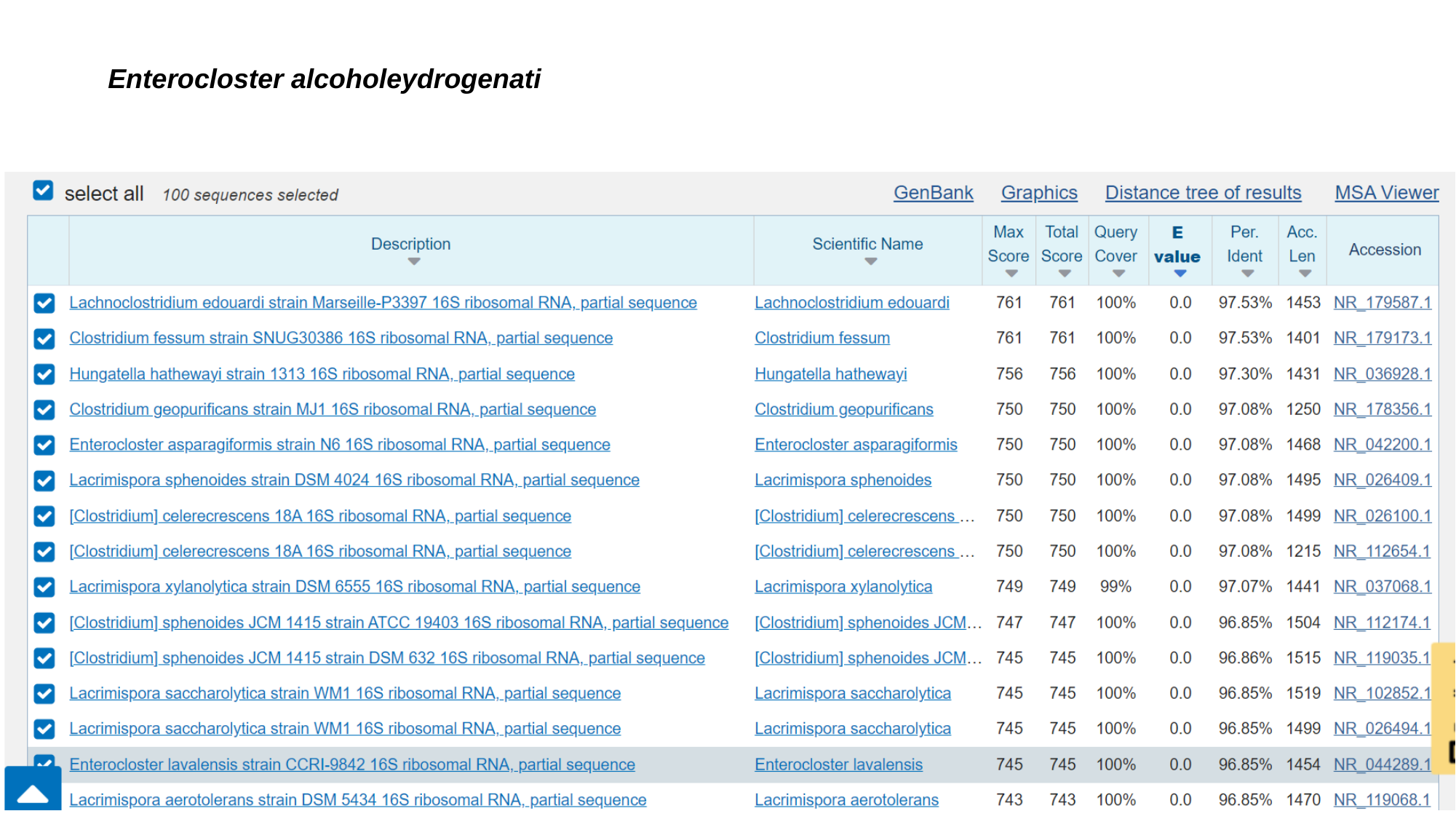

Enterocloster alcoholeydrogenati
