# Supplementary material for: Increased fecal ethanol and enriched ethanol-producing gut bacteria Limosilactobacillus fermentum, Enterocloster bolteae, Mediterraneibacter gnavus and Streptococcus mutans in nonalcoholic steatohepatitis
Source: Front Cell Infect Microbiol. 2023 Nov 16;13:1279354. doi: 10.3389/fcimb.2023.1279354 (PMC10687429; doi:10.3389/fcimb.2023.1279354)

**Increased Fecal Ethanol and Enriched Ethanol-producing Gut Bacteria *Limosilactobacillus fermentum*, *Enterocloster bolteae*, *Mediterraneibacter gnavus* and *Streptococcus mutans* in Nonalcoholic Steatohepatitis**

Babacar MBAYE, Reham MAGDY WASFY, Patrick BORENTAIN, Maryam TIDJANI ALOU, Giovanna MOTTOLA, Vincent BOSSI, Aurelia CAPUTO, Rene GEROLAMI, Matthieu MILLION

**SUPPLEMENTARY DATA**

**Supplementary table 1. Biochemical results**

See Excel file, sheet TableS1

**Supplementary Table 2. Culturomics results**

See Excel file, sheet TableS2

**Supplementary Table 3. V3V4 16S metagenomics results**

See Excel file, sheet TableS3

**Supplementary Table 4. 16S metagenomics results after blastN**

**Supplementary Table 4. Operational taxonomy units corresponding to several species**

| **Most probable species according to culturomics (viable bacteria found in human fecal samples in present or previous culturomics study)** | **All possible species corresponding to this OTU** | **Reference** |
| --- | --- | --- |
| Streptococcus_salivarius_OTU1 | Streptococcus_salivarius / Streptococcus_vestibularis | Delorme C, Abraham AL, Renault P, Guédon E. Genomics of Streptococcus salivarius, a major human commensal. Infect Genet Evol. 2015;33:381-392. doi:10.1016/j.meegid.2014.10.001 |
| Blautia_wexlerae_OTU2 | Blautia_maliae / Blautia_provencensis / Blautia_timonensis / Blautia_wexlerae | Liu C, Finegold SM, Song Y, Lawson PA. Reclassification of Clostridium coccoides, Ruminococcus hansenii, Ruminococcus hydrogenotrophicus, Ruminococcus luti, Ruminococcus productus and Ruminococcus schinkii as Blautia coccoides gen. nov., comb. nov., Blautia hansenii comb. nov., Blautia hydrogenotrophica comb. nov., Blautia luti comb. nov., Blautia producta comb. nov., Blautia schinkii comb. nov. and description of Blautia wexlerae sp. nov., isolated from human faeces. Int J Syst Evol Microbiol. 2008;58(Pt 8):1896-1902. doi:10.1099/ijs.0.65208-0 |
| Limosilactobacillus_caccae_OTU3 | Lactobacillus_antri / Lactobacillus_caccae / Lactobacillus_frumenti / Lactobacillus_oris / Lactobacillus_panis / Lactobacillus_vaginalis | Lo CI, Dione N, Mbaye A, et al. Limosilactobacillus caccae sp. nov., a new bacterial species isolated from the human gut microbiota [published correction appears in FEMS Microbiol Lett. 2023 Jan 17;370:]. FEMS Microbiol Lett. 2021;368(18):fnab128. doi:10.1093/femsle/fnab128 |
| Streptococcus_timonensis_OTU4 | Streptococcus_infantis / Streptococcus_massilioralis / Streptococcus_mitis / Streptococcus_oralis / Streptococcus_pneumoniae / Streptococcus_pseudopneumoniae / Streptococcus_timonensis / Streptococcus_urinomassiliensis | Ricaboni D, Mailhe M, Lagier JC, et al. Noncontiguous finished genome sequence and description of Streptococcus timonensis sp. nov. isolated from the human stomach. New Microbes New Infect. 2016;15:77-88. Published 2016 Nov 18. doi:10.1016/j.nmni.2016.11.013 |
| Bifidobacterium_adolescentis_OTU5 | Bifidobacterium_adolescentis / Bifidobacterium_catenulatum / Bifidobacterium_faecale / Bifidobacterium_kashiwanohense / Bifidobacterium_pseudocatenulatum / Bifidobacterium_ruminantium | Duranti S, Ruiz L, Lugli GA, et al. Bifidobacterium adolescentis as a key member of the human gut microbiota in the production of GABA. Sci Rep. 2020;10(1):14112. Published 2020 Aug 24. doi:10.1038/s41598-020-70986-z |
| Streptococcus_anginosus_OTU7 | Streptococcus_anginosus / Streptococcus_intermedius | Masood U, Sharma A, Lowe D, Khan R, Manocha D. Colorectal Cancer Associated with Streptococcus anginosus Bacteremia and Liver Abscesses. Case Rep Gastroenterol. 2016;10(3):769-774. Published 2016 Dec 13. doi:10.1159/000452757 |
| Actinomyces_naeslundii_OTU8 | Actinomyces_oralis / Actinomyces_bowdenii / Actinomyces_johnsonii / Actinomyces_naeslundii / Actinomyces_oris / Actinomyces_viscosus | Cisar JO, Kolenbrander PE, McIntire FC. Specificity of coaggregation reactions between human oral streptococci and strains of Actinomyces viscosus or Actinomyces naeslundii. Infect Immun. 1979;24(3):742-752. doi:10.1128/iai.24.3.742-752.1979 |
| Lactobacillus_gasseri_OTU9 | Lactobacillus_gasseri / Lactobacillus_hominis / Lactobacillus_taiwanensis | Azcarate-Peril MA, Altermann E, Goh YJ, et al. Analysis of the genome sequence of *Lactobacillus gasseri* ATCC 33323 reveals the molecular basis of an autochthonous intestinal organism. Appl Environ Microbiol. 2008;74(15):4610-4625. doi:10.1128/AEM.00054-08 |
| Streptococcus_sinensis_OTU10 | Streptococcus_boccae / Streptococcus_sinensis | Woo PC, Tam DM, Leung KW, et al. *Streptococcus sinensis* sp. nov., a novel species isolated from a patient with infective endocarditis. J Clin Microbiol. 2002;40(3):805-810. doi:10.1128/JCM.40.3.805-810.2002 |
| Gemella_haemolysans_OTU11 | Gemella_haemolysans / Gemella_sanguinis | Kodaka S, Uchida T, Gomi H. *Gemella haemolysans* as an emerging pathogen for bacteremia among the elderly. J Gen Fam Med. 2021;23(2):110-112. Published 2021 Oct 5. doi:10.1002/jgf2.497 |
| Streptococcus_peroris_OTU12 | Streptococcus_lactarius / Streptococcus_peroris / Streptococcus_sinensis | Kawamura Y, Hou XG, Todome Y, et al. *Streptococcus peroris* sp. nov. and *Streptococcus infantis* sp. nov., new members of the *Streptococcus mitis* group, isolated from human clinical specimens. Int J Syst Bacteriol. 1998;48 Pt 3:921-927. doi:10.1099/00207713-48-3-921 |
| Veillonella_parvula_OTU13 | Veillonella_parvula / Veillonella_rogosae / Veillonella_tobetsuensis | Veillon A, Zuber A. Recherches sur quelques microbes strictement anaérobies et leur rôle en pathologie. Archives de Mèdecine Expérimentale et d'Anatomie Pathologique 1898; 10:517-545. |
| Bacteroides_faecis_OTU14 | Bacteroides_faecis / Bacteroides_finegoldii | Kim MS, Roh SW, Bae JW. *Bacteroides faecis* sp. nov., isolated from human faeces. Int J Syst Evol Microbiol. 2010;60(Pt 11):2572-2576. doi:10.1099/ijs.0.020024-0 |
| Bacteroides_caccae_OTU15 | Bacteroides_caccae / Bacteroides_transplantocaccae | Johnson JL, Moore WEC, Moore LVH*. Bacteroides caccae* sp. nov., *Bacteroides merdae* sp. nov., and *Bacteroides stercoris* sp. nov. isolated from human feces. Int. J. Syst. Bacteriol. 1986 ; 36:499-501 |
| Oscillibacter_caccae_OTU16 | Oscillibacter_caccae / Oscillibacter_mediterraneensis | Under description |
| Hungatella_hathewayi_OTU17 | Hungatella_effluvii / Hungatella_hathewayi | Steer T, Collins MD, Gibson GR, Hippe H, Lawson PA. *Clostridium hathewayi* sp. nov., from human faeces. Syst Appl Microbiol. 2001;24(3):353-357. doi:10.1078/0723-2020-00044 |
| Bacteroides_intestinalis_OTU18 | Bacteroides_cellulosilyticus / Bacteroides_intestinalis / Bacteroides_timonensis | Bakir MA, Kitahara M, Sakamoto M, Matsumoto M, Benno Y. *Bacteroides intestinalis* sp. nov., isolated from human faeces. Int J Syst Evol Microbiol. 2006;56(Pt 1):151-154. doi:10.1099/ijs.0.63914-0 |
| Tidjanibacter_massiliensis_OTU19 | Alistipes_inops / Tidjanibacter_massiliensis | Mailhe M, Ricaboni D, Benezech A, Lagier JC, Fournier PE, Raoult D. *'Tidjanibacter massiliensis'* gen. nov., sp. nov., a new bacterial species isolated from human colon. New Microbes New Infect. 2016;17:21-22. Published 2016 Dec 13. doi:10.1016/j.nmni.2016.12.010 |

| **Species** | **Culturomics** | **Frequency difference before blast all samples** | **Frequency difference after blast all samples** |
| --- | --- | --- | --- |
| *Bacteroides thetaiotaomicron* |  |  |  |
| *Cutibacterium eggermontii* |  |  |  |
| *Enterocloster bolteae* |  |  |  |
| *Enterococcus avium* |  |  |  |
| *Facklamia hominis* |  |  |  |
| *Finegoldia magna* |  |  |  |
| *Holdemanella biformis* |  |  |  |
| *Limosilactobacillus fermentum* |  |  |  |
| *Peptoniphilus grossensis* |  |  |  |
| *Peptoniphilus koenoeneniae* |  |  |  |
| *Streptococcus constellatus* |  |  |  |
| *Alistipes obesi* |  |  |  |
| *Lacticaseibacillus casei* |  |  |  |
| *Lentilactobacillus parabuchneri* |  |  |  |
| *Phascolarctobacterium faecium* |  |  |  |

**Supplementary Table 5. Comparaison between culturomics species and 16S datasets**

**Figure S1. Alpha and beta diversity results of 16S metagenomics results assessed by the Microbiome analyst pipeline**


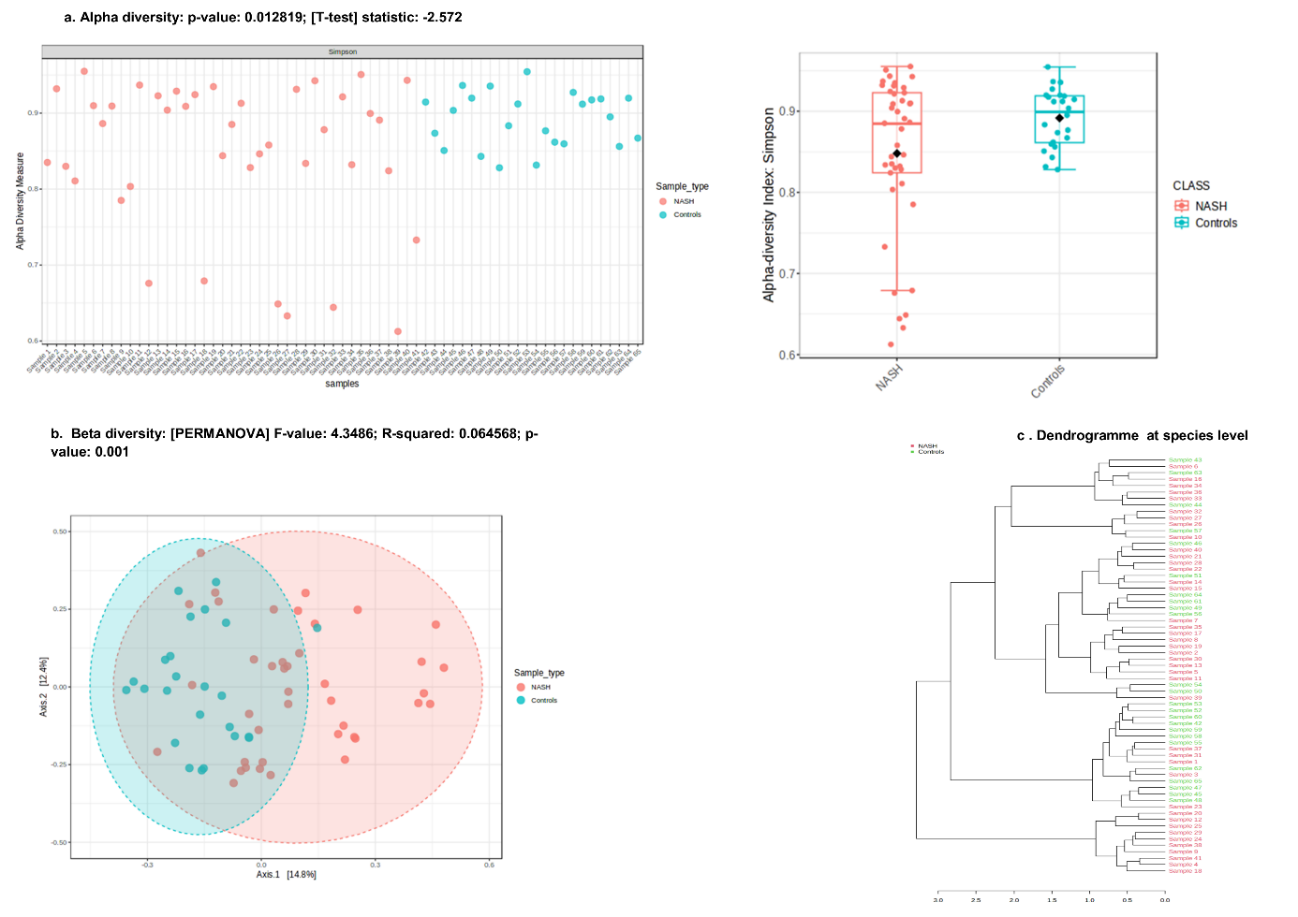


a. Apha diversity Simpson index was lower in NASH patients. Strikingly, 7 NASH patients had very low Simpson indexes. b. Beta-diversity evidenced an increased heterogeneity of NASH samples compared to controls. c. Dendrogram at the species level did not evidenced a clear distinction between cases and controls.

**Figure S2. Linear discriminant analysis (LDA) between 41 NASH and 24 Healthy controls using the Microbiome analyst pipeline (Phylum, Family and Genus level)**

**
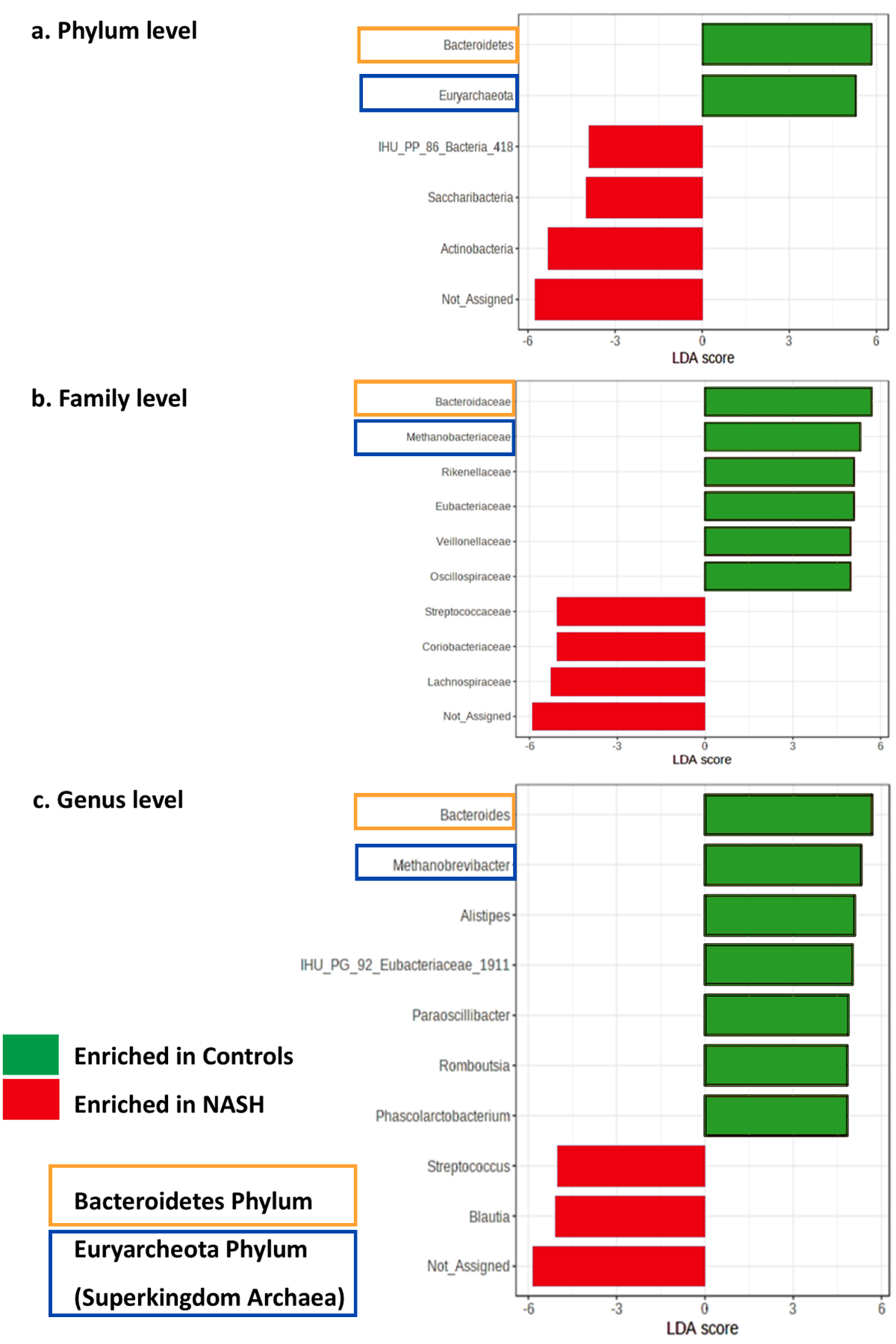
**

The Microbiome analyst pipeline is available online (<https://www.microbiomeanalyst.ca/MicrobiomeAnalyst/home.xhtml>).

**Figure S3. Linear discriminant analysis (LDA) between 41 NASH and 24 Healthy controls using the Microbiome analyst pipeline (Species level)**

**
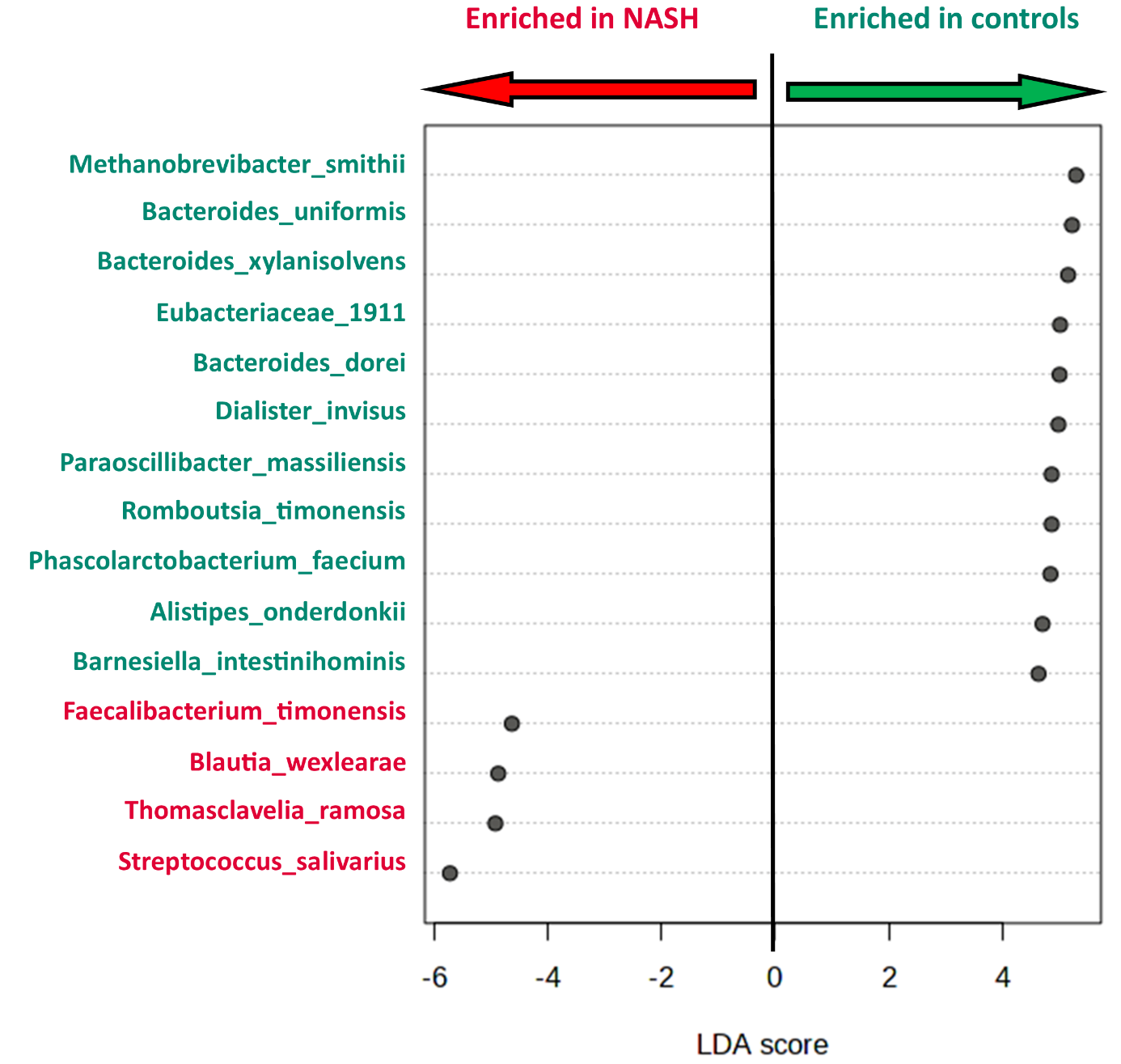
**

**Figure S4. Frequency difference of Species enriched in culture after blast analyses datasets**

**41NASH and 24 controls**

**Figure S5. Frequency difference of Species enriched in culture before blast analyses datasets**

**41 NASH and 24 controls**


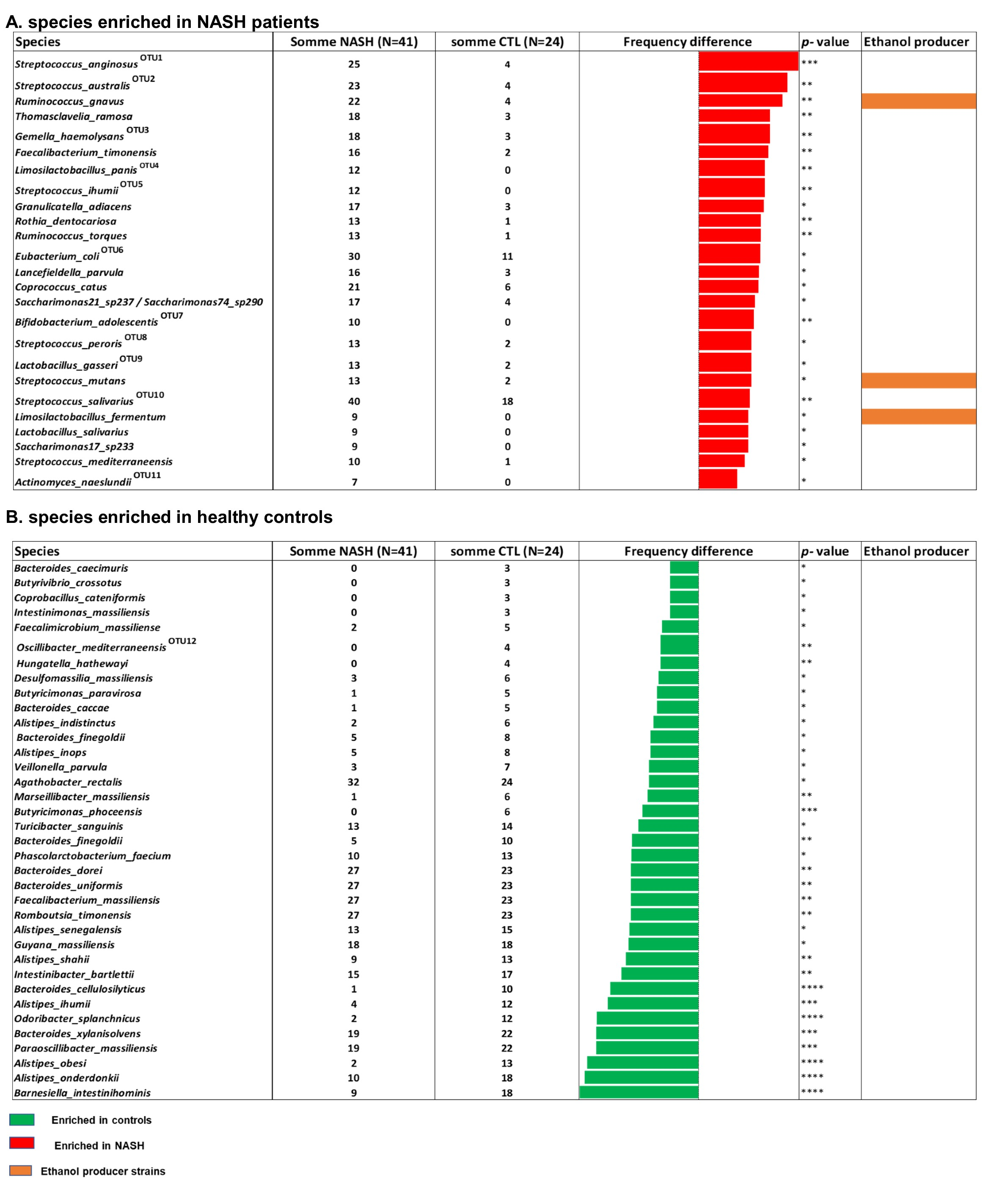

Supplement: Supplementary file 2 [file Table_1.docx]
